# Estimating age-stratified influenza-associated invasive pneumococcal disease in England: A time-series model based on population surveillance data

**DOI:** 10.1371/journal.pmed.1002829

**Published:** 2019-06-27

**Authors:** Chiara Chiavenna, Anne M. Presanis, Andre Charlett, Simon de Lusignan, Shamez Ladhani, Richard G. Pebody, Daniela De Angelis

**Affiliations:** 1 MRC Biostatistics Unit, School of Clinical Medicine, University of Cambridge, Cambridge, United Kingdom; 2 National Infection Service, Public Health England, London, United Kingdom; 3 Department of Clinical and Experimental Medicine, University of Surrey, Surrey, United Kingdom; Edinburgh University, UNITED KINGDOM

## Abstract

**Background:**

Measures of the contribution of influenza to *Streptococcus pneumoniae* infections, both in the seasonal and pandemic setting, are needed to predict the burden of secondary bacterial infections in future pandemics to inform stockpiling. The magnitude of the interaction between these two pathogens has been difficult to quantify because both infections are mainly clinically diagnosed based on signs and symptoms; a combined viral–bacterial testing is rarely performed in routine clinical practice; and surveillance data suffer from confounding problems common to all ecological studies. We proposed a novel multivariate model for age-stratified disease incidence, incorporating contact patterns and estimating disease transmission within and across groups.

**Methods and findings:**

We used surveillance data from England over the years 2009 to 2017. Influenza infections were identified through the virological testing of samples taken from patients diagnosed with influenza-like illness (ILI) within the sentinel scheme run by the Royal College of General Practitioners (RCGP). Invasive pneumococcal disease (IPD) cases were routinely reported to Public Health England (PHE) by all the microbiology laboratories included in the national surveillance system. IPD counts at week *t*, conditional on the previous time point *t*−1, were assumed to be negative binomially distributed. Influenza counts were linearly included in the model for the mean IPD counts along with an endemic component describing some seasonal background and an autoregressive component mimicking pneumococcal transmission. Using age-specific counts, Akaike information criterion (AIC)-based model selection suggested that the best fit was obtained when the endemic component was expressed as a function of observed temperature and rainfall. Pneumococcal transmission within the same age group was estimated to explain 33.0% (confidence interval [CI] 24.9%–39.9%) of new cases in the elderly, whereas 50.7% (CI 38.8%–63.2%) of incidence in adults aged 15–44 years was attributed to transmission from another age group. The contribution of influenza on IPD during the 2009 pandemic also appeared to vary greatly across subgroups, being highest in school-age children and adults (18.3%, CI 9.4%–28.2%, and 6.07%, CI 2.83%–9.76%, respectively). Other viral infections, such as respiratory syncytial virus (RSV) and rhinovirus, also seemed to have an impact on IPD: RSV contributed 1.87% (CI 0.89%–3.08%) to pneumococcal infections in the 65+ group, whereas 2.14% (CI 0.87%–3.57%) of cases in the group of 45- to 64-year-olds were attributed to rhinovirus. The validity of this modelling strategy relies on the assumption that viral surveillance adequately represents the true incidence of influenza in the population, whereas the small numbers of IPD cases observed in the younger age groups led to significant uncertainty around some parameter estimates.

**Conclusions:**

Our estimates suggested that a pandemic wave of influenza A/H1N1 with comparable severity to the 2009 pandemic could have a modest impact on school-age children and adults in terms of IPD and a small to negligible impact on infants and the elderly. The seasonal impact of other viruses such as RSV and rhinovirus was instead more important in the older population groups.

## Abstract 2

Using surveillance data from England between 2009 and 2017, Chiara Chiavenna and colleagues estimate the contribution of influenza infection on invasive pneumococcal disease.

## Author summary

**Why was this study done?:**

- Since the deadly 1918 Spanish flu, pandemic preparedness has been crucial for many governments: in the United Kingdom, a pandemic is high on the government's national risk register of civil emergencies [1].
- Previous research has shed light on the central role of secondary bacterial infections, suggesting a synergistic interplay of influenza virus and *S*. *pneumoniae*, a bacterium that infects the lungs.
- Quantifying the magnitude of such interaction at the population level is of central importance to inform public health policy: in England, current decision-making on required sizes of an antibiotic stockpile for use in a future pandemic lacks scientific evidence.

**What did the researchers do and find?:**

- We analysed routinely collected population-level counts of invasive pneumococcal disease (IPD) cases.
- We decomposed the incidence of IPD in terms of endemic pneumococcal circulation, a seasonal contribution, and an influenza-driven component.
- We proposed a novel multivariate age-stratified modelling framework to assess the contribution of influenza across seasons and age groups.

**What do these findings mean?:**

- We found that influenza played a more important role towards explaining IPD during the 2009 pandemic than during seasonal epidemics.
- This role was particularly prominent in school-age children.
- These results are valuable to quantify the possible contribution of influenza to the burden of IPD in a future pandemic of influenza with similar characteristics to the 2009 pandemic.

## Introduction

Just one century ago, the "1918 Spanish Influenza" is thought to have caused at least 50 million deaths worldwide despite influenza often naively being considered to be a nonsevere disease. Hence, a number of researchers in recent decades have tried to understand the drivers of such severity in the fear of a new pandemic [2–4]. Viral–bacterial synergism, in particular with *S*. *pneumoniae*, is considered to have played a major role in the observed mortality rate, as postmortem examinations revealed the presence of bacteria in the lungs of many influenza-infected individuals [5].

The synergistic interplay between influenza and *S*. *pneumoniae* has been validated in animal models [6]; however, routine ascertainment of coinfection remains difficult and expensive in humans [7]: individual-level data on the exposure are hard to acquire because pathogens often circulate silently within a host population or manifest themselves through nonspecific clinical symptoms [8–10]. Infections due to each pathogen are separately identified in the presence of a corresponding disease, and the likelihood of an improved understanding of pathogen interactions strongly relies on indirect inference.

Time series of respiratory diseases are characterised by strong seasonal patterns, with an increased incidence in the winter months in temperate areas of the world. Disentangling the contribution towards *S*. *pneumoniae* of the influenza virus from other risk factors that exhibit the same seasonal variation (e.g., weather, daylight, circulation of other pathogens, etc.) [11] can be challenging. A variety of regression methods have been suggested in the general framework of burden estimation, especially for excess morbidity and mortality due to seasonal and pandemic influenza [12, 13].

Using sentinel testing of suspected influenza cases, the presence and magnitude of influenza virus in the community is usually summarised by the proportion of positive tests by viral type (and/or subtype). The so-called virological regression model includes influenza circulation as a covariate in a cyclic regression model for respiratory infections, in which seasonality of disease is described by sine and cosine terms [14, 15]. Lagged effects of virological circulation, or other confounders that exhibit annual variation in intensity or timing, can also be included [16]. The most adequate distribution for the outcome variable has been widely debated: [17] argued that counts of disease should be modelled as Poisson distributed, employing a log-link function; however, such a link implies an exponential increase of the outcome with respect to the number of confirmed influenza cases and multiplicative effects of covariates (i.e., respiratory viruses). As these assumptions are quite unrealistic, [18] and [19] suggested the use of a generalized linear model (GLM) with a Poisson error distribution but identity link [20, 21].

Previous work estimated the burden of influenza on syndromic healthcare contacts, such as lower respiratory tract infection (LRTI) [3], acute respiratory illness (ARI) [22], or respiratory hospital admissions [23]; however, this has not elucidated the relative contribution of the interaction between influenza virus and *S*. *pneumoniae* relative to shared seasonality [3, 24, 25]. Reference [26] estimated the percentage of invasive pneumococcal disease (IPD) cases attributable to influenza and respiratory syncytial virus (RSV) using regression models; however, since we are dealing with a transmissible pathogen, the independence among observations they assume is unlikely to hold. Autoregressive integrated moving average (ARIMA) models have also been proposed [27]; however, such an approach and its multivariate counterpart, ARIMAX, require applying preliminary transformations to the original data when nonstationary behaviour is detected. The necessity of choosing model order via an empirical procedure based on model fit, along with the limited interpretability of coefficients, precludes ARIMA methods as a sensible choice for our scope [28].

We combined the key strengths of each of the previous approaches into a single flexible regression model, following the work of [29]: weekly IPD counts were decomposed into an endemic component, with sine–cosine waves describing cyclic winter outbreaks, and an epidemic autoregressive component, in which lagged IPD counts entered the model linearly using an identity link function [30]. A time-varying covariate could also be linearly added to the model, with the corresponding coefficient expressing the association between the two time series after taking into account shared drivers [31].

We extended the modelling framework of [29] to address a number of issues. First, we were interested in investigating the contribution of several pathogens to the incidence of IPD: other viruses such as RSV and rhinovirus have been speculated to interact with *S*. *pnuemoniae*, showing an association with an increased risk of IPD [32, 33]. In contrast to this existing work, we jointly modelled the epidemic evolution of viruses of interest by simultaneously including them as covariates in models of the IPD counts. Secondly, associations between pathogens have been suggested to be heterogeneous across age groups [34]; hence, we implemented multivariate versions of the model allowing estimation of age-specific associations. The multivariate structure also permitted decomposition of IPD transmission between and across age groups by incorporating contact patterns. Finally, as there is evidence that meteorological conditions such as temperature and humidity affect seasonality and intensity of outbreaks [35, 36], we replaced sinusoidal functions with observed weather information. Compared to previous work [37, 38], we proposed a phenomenological model that expresses IPD dynamics as a function of autoregressive components, viral infections, age-specific contact patterns, and seasonal confounders without making strong assumptions on the transmission mechanism, aiming to provide a parsimonious characterisation of the drivers of IPD patterns over time.

## Methods

### Data

Influenza is generally diagnosed based on influenza-like illness (ILI), defined as the simultaneous presence of signs and symptoms such as high fever, cough, and myalgia; however, only virological testing allows the ascertainment of the responsible pathogen. For this reason, we estimated influenza incidence by combining two data sources. The Royal College of General Practitioners Research and Surveillance Centre (RCGP RSC) collects weekly numbers of general practice consultations for several clinical diagnoses of communicable and respiratory diseases, including ILI. The population monitored by the RCGP RSC practices covers an average population of approximately 1.4 million persons, 2.6% of England, considered to be representative of the national population in terms of age, gender, deprivation index, and prescription patterns [39]. As part of routine virological surveillance, in general practices participating in the RCGP RSC scheme, a proportion of ILI cases is swabbed and the samples are tested for influenza A (H1 or H3 subtypes), influenza B, RSV, and human metapneumovirus (hMPV) by the Public Health England (PHE) reference laboratory [39]. The number of specimens tested and the number of positives for each virus were stratified by week of test and age group to derive the proportion of virologically positive specimens. This proportion was then multiplied by ILI counts to compute the corresponding age and time-specific consultations attributable to influenza.

*S*. *pneumoniae* (the pneumococcus) infection is often asymptomatic, as this is a commensal bacterium of the human nasopharynx; nonetheless, its progression to the lower respiratory tract and blood can cause severe disease, namely IPD. In the UK, counts of positive isolates for a number of clinically significant pathogens are reported weekly to PHE by all the microbiology laboratories included in the national surveillance system and are stored in the Second Generation Surveillance System (SGSS) database. Counts of IPD, RSV, and rhinovirus infections were extracted from SGSS. Consistency in testing over time and space was guaranteed by the ‘United Kingdom Standards for Microbiology Investigations’, a diagnostic algorithm applied across laboratories to patients presenting with different clinical syndromes [40]. Finally, estimates of the population of England by age group, during each season, were obtained from the Office for National Statistics [41], and weather information such as daily central England temperature and daily England and Wales precipitation were downloaded from the MetOffice HadCET data repository [42].

This study is reported as per the Guidelines for Accurate and Transparent Health Estimates Reporting (GATHER) [43] (S1 Checklist). The study did not have a prospective design, and analysis was planned as we retrospectively gathered information on routinely collected and publicly available data sources, considering England as being representative of temperate areas in the northern hemisphere, where the time series of interest feature typical winter peaks. The time period considered ranged from 1 January 2009 to 31 December 2017, with the 2009 pandemic period defined to include the three waves, from week 15/2009 to week 26/2011 [44]. Disease incidence was categorised into five age groups: 0–4, 5–14, 15–44, 45–64, and 65+ years old, as in similar studies [26].

### Statistical model

When dealing with two strongly associated time-series representing infectious disease incidence, the modelling framework presented by [31] allows quantification of the relationship between outbreaks of the two pathogens of interest. Denoting by *Y*_*t*_ the random variables representing counts of disease *Y* at weeks *t* = 1,…,*T*, it is assumed they are Poisson distributed: *Y*_*t*_|*Y*_*t*−1_~*Poi*(*μ*_*t*_), with conditional mean *μ*_*t*_ expressed as

$$\mu_{t}=pop_{t}\nu_{t}+\lambda Y_{t-1}+\tau X_{t-1},$$ (1)

where *ν*_*t*_ is an endemic log-linear predictor that, multiplied by an offset such as population size *pop*_*t*_, might describe incidence due to seasonal or sociodemographic variation; *Y*_*t*−1_ is a temporal interaction (epidemic component) whose coefficient *λ* represents the transmission of infection from time *t*−1 to time *t*; and *X*_*t*−1_ are lagged counts of disease *X*, with the coefficient *τ* quantifying the strength of association between *Y*_*t*_ and *X*_*t*−1_. For overdispersed counts, the Poisson distribution for the observation model can be replaced by a negative binomial with overdispersion parameter *ψ*:

$$Y_{t}|Y_{t-1}∼NegBin(\mu_{t},\psi ).$$ (2)

The decomposition of the contribution of several phenomena in additive components, along with the small number of parameters, makes interpretation very straightforward while preserving biologically meaningful relationships among the quantities of interest. Moreover, compared to the parameter-driven models briefly reviewed in the introduction that are characterised by harmonic functions, the presence of an observation-driven component in model (1) could capture outbreaks more easily, as *λ* expresses the additional temporal dependence beyond the seasonality explained by the parametric model [45]. Finally, modelling overdispersion instead of assuming Poisson-distributed outcomes allows further flexibility.

The model in Eq 1 can be easily extended to deal with stratified time series: [46] implemented a multivariate version for spatial disease spread and later embedded an age-structure into it [47]. We followed a similar approach when modelling disease counts in age group *a*, *Y*_*a*,*t*_, where *a*∈ {0–4, 5–14, 15–44, 45–64, 65+}. Two transmission components were included at this stage:

$$\mu_{t,a}=pop_{t,a}\nu_{t,a}+\lambda_{a}Y_{t-1,a}+\phi_{a}\sum_{k\neq a}c_{k,a}Y_{t-1,k\neq a}+\tau_{a}X_{t-1,a}.$$ (3)

In addition to the transmission of one pathogen within age group *a*, quantified by *λ*_*a*_, we explicitly incorporated the transmission of the same pathogen across age groups through *ϕ*_*a*_. In order to account for heterogeneity of contact patterns, counts of disease in groups *k*≠*a* were weighted by the element *c*_*k*,*a*_ of a contact matrix (e.g., POLYMOD or any other measure of social distancing between group *k* and *a* [48]). Hence, the coefficient *ϕ*_*a*_, paired with such a linear combination of disease cases, represents the contribution of transmission from other population subgroups to disease in age group *a*. Both transmission coefficients were specified to be age-specific because, despite accounting for contact patterns, some age groups are known to be more susceptible to infection than others. We also allowed heterogeneity across groups for the remaining model parameters, as the interaction between influenza and *S*. *pneumoniae* has also been suggested to vary with age [7]. Finally, we extended this setting to incorporate more than two time series, estimating the association of the outcome of interest with more than one pathogen (e.g., other indicators of viral circulation such as rhinovirus and RSV incidence).

Models in Eqs 1 and 2 are both implemented in the R package ‘surveillance’ through the hhh4 function. For the model in Eq 3, in which multiple covariates were added, our algorithm simultaneously fitted models for different strata incorporating the contact structure. Similarly to the hhh4 function, we also obtained maximum-likelihood estimates via a (globally convergent) Newton-Raphson type algorithm. To ensure positivity, parameters were optimised on the log-scale—i.e., *log*(*ψ*) and *log*(*λ*) were used. Uncertainty about the proportions of IPD cases attributable to each virus was estimated by resampling n = 10,000 datasets from the fitted model and taking the 95% confidence intervals (CIs) to be the empirical 2.5% and 97.5% percentiles across the resampled datasets.

## Results

**Fig 1:**
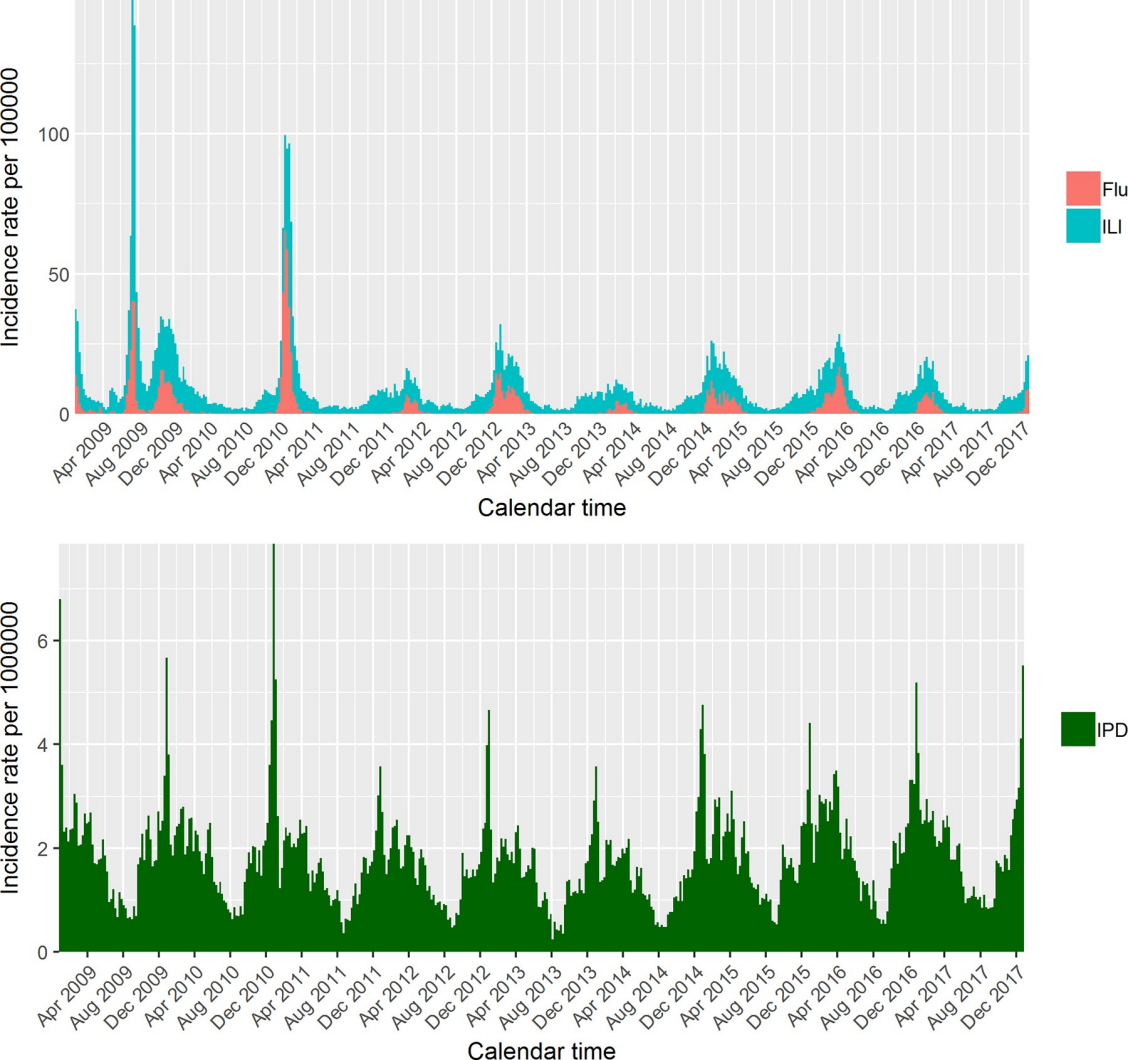
ILI and Flu incidence rate in the top panel; IPD incidence rate in the bottom panel. ILI, influenza-like illness; IPD, invasive pneumococcal disease.

A total of 62,679 ILI consultations within the sentinel scheme and of 45,601 IPD cases nationwide have been notified over 9 years. Fig 1 displays the temporal trend of all ILI and influenza-confirmed consultation rates respectively, where influenza-confirmed counts (referred to as ‘Flu’ from now on) were obtained as described in the Methods. A clear seasonal pattern is visible, with regular outbreaks in the winter months and epidemics lasting 10–15 weeks, except for 2009, when the A/H1N1 pandemic started in spring. Virological testing is not systematically performed during the summer; hence, the Flu data are quite sparse off-season. Nonetheless, it is evident how, even during winter, the influenza cases do not closely mimic the ILI curve, confirming the nonspecificity of the ILI diagnosis. In the IPD time series (Fig 1, bottom panel), peaks appear to be similar across seasons both in terms of amplitude and timing, with a gradual increase of cases from autumn to a winter peak, followed by a decline in summer. The incidence rate per 1,000,000 population is plotted in this case, as IPD is rare.

We followed the analysis strategy reported in full in the S1 Appendix. Briefly, the best formulation for the model in Eq 1 was first identified in terms of Akaike information criterion (AIC) values. A summary of model comparison is presented in Table 1: starting from a Poisson distributional assumption and one set of harmonic functions (*S* = 1, see S1 Appendix), more complicated versions of the endemic component were assessed by replacing trigonometric waves with weather variables (model C). We also tested whether multiple lags for covariates better described the observed patterns: considering lags *q* = 1,…,*Q* where *Q* = 5—i.e., including up to 5 weeks before time *t*, we saw no gain in adding either Flu or IPD lagged counts when *q*>1. The only variables whose lagged values improved model fit were rainfall and temperature; nonetheless, the parameter representing the decline in weight attributed to lagged values was optimally chosen to be *p*_*weather*_ = 0.8, suggesting that only 20% of the weight is attributed to observations more than one week before (model D).

**Table 1:** Model comparison in terms of AIC and one-step-ahead forecast (log[s(P,x)]).

	Distribution	Endemic	Covariate	AIC	log(s[P,x])
A	Poi	S = 1	Flu	5,107.61	5.805
B	NB	S = 1	Flu	4,043.95	4.408
C	NB	rain + temp, lag = 1	Flu	4,029.19	4.400
D	NB	rain + temp, lags = 5 (*p*_*weather*_ = 0.8)	Flu	4,027.82	4.390
E	NB	rain + temp, lags = 5 (*p*_*weather*_ = 0.8)	Flu + rhinov	3,997.93	4.361
F	NB	rain + temp, lags = 5 (*p*_*weather*_ = 0.8)	Flu + rhinov + RSV	3,992.95	4.334

Abbreviations: AIC, Akaike information criterion; NB, negative binomial; Poi, Poisson; rhinov, rhinovirus; RSV, respiratory syncytial virus; temp, temperature

Evaluating the model in terms of one-step-ahead forecasts, we selected 30 weeks as the initial time window of observed data, and we repeated the forecast for each of the remaining 440 weeks. We reassuringly found mean *log*(*s*(*P*,*x*)) (see S1 Appendix) to be minimal for the endemic formulation including weather information, with lags weighted according to *p*_*weather*_ = 0.8 (model D).

**Fig 2:**
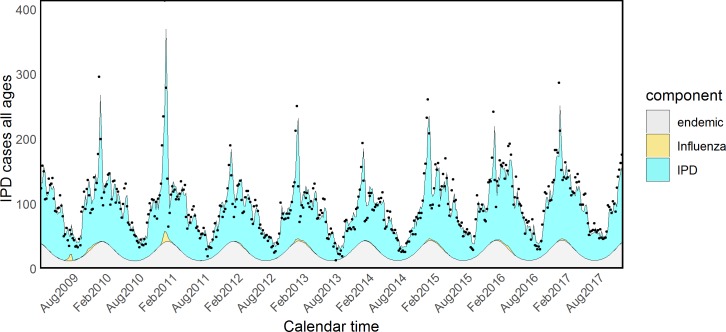
Model (B) of IPD and influenza with one set of harmonic functions. IPD, invasive pneumococcal disease.

**Fig 3:**
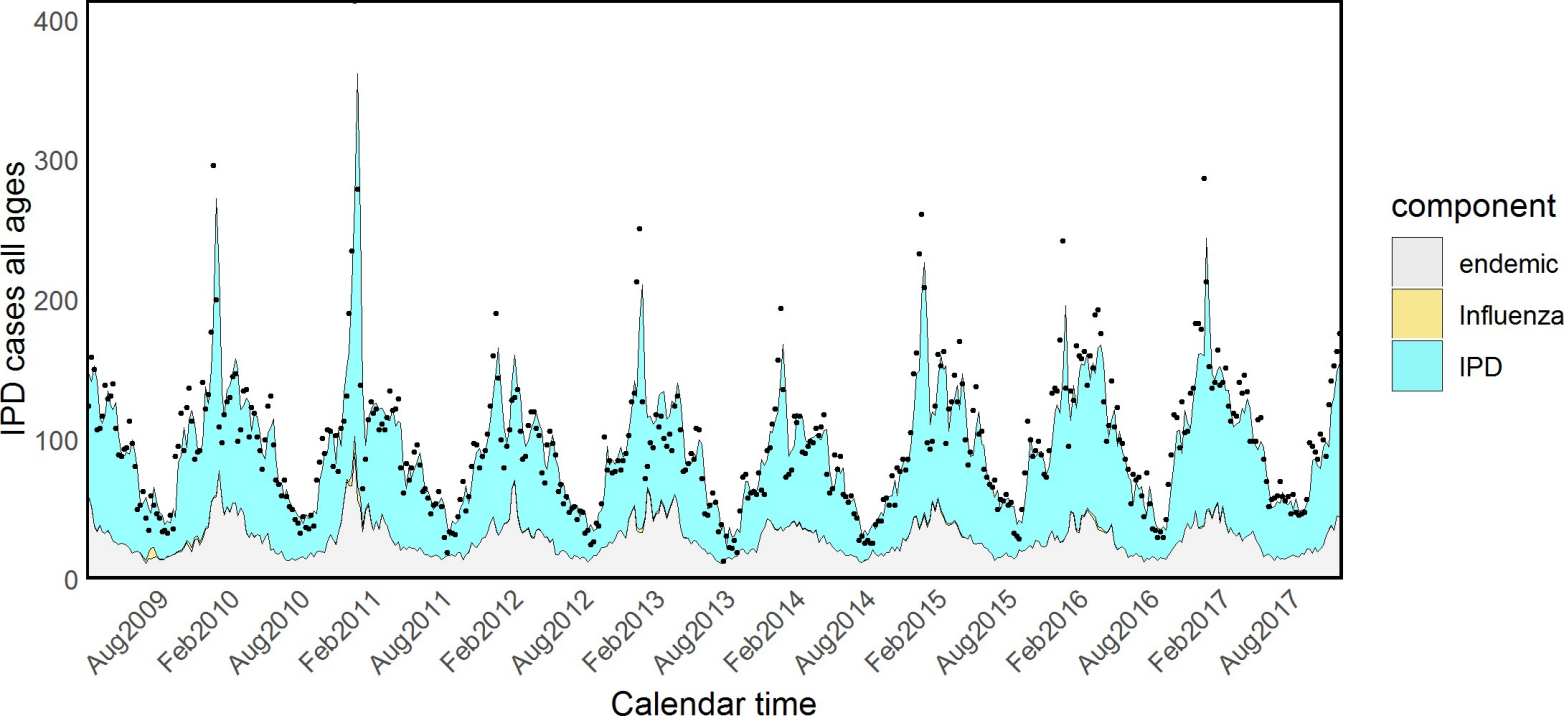
Model (E) of IPD and influenza with rainfall and temperature. IPD, invasive pneumococcal disease.

Fitted values for all components according to model formulations B and D are shown in Figs 2 and 3. The number of IPD cases attributed to Flu during the entire study period was as low as 199 according to model D, including weather variables—i.e., 0.45% (CI < 0.01%–1.59%) of all the IPD cases. However, 100 of these cases happened during the three pandemic waves, 0.83%, CI < 0.01%–2.94%, of all the observed IPD cases in that period, suggesting that the pandemic strain might have been responsible for an increased incidence. As a sensitivity analysis, we selected Flu counts referring only to the three pandemic waves: the increase in AIC was minimal compared to model D including Flu counts over all the study period, suggesting that the role of seasonal Flu is marginal. We also considered each season as a separate covariate, with results plotted in S1 Fig.

**Fig 4:**
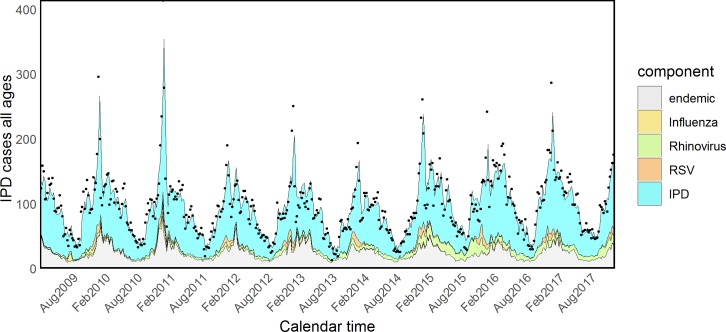
Model (F) including influenza, rhinovirus, and RSV. IPD, invasive pneumococcal disease; RSV, respiratory syncytial virus.

Finally, we investigated whether other viruses also interact with *S*. *pneumoniae*: the number of rhinovirus (model E in Table 1) and RSV (model F) infections were sequentially added to the selected model D (the observed time series are plotted in S2 and S3 Figs). Rhinovirus alone greatly enhanced the fit to the data, and the inclusion of RSV on top of Flu and rhinovirus still resulted in model improvement. Hence, the best-fitting model (F) for mean IPD counts at time *t* takes the form

$$\begin{array}{c} \mu_{IPD,t}=pop_{t}[exp(\alpha +\gamma \sum_{q=1}^{5}w_{q}(weather)temp_{t-q}+\delta \sum_{q=1}^{5}w_{q}(weather)rain_{t-q})]+\\ +\lambda IPD_{t-1}+\tau Flu_{t-1}+\theta rhinovirus_{t-1}+\zeta RSV_{t-1} \end{array}$$ (4)

with overdispersion parameter *ψ* and decay parameter for *w*_*q*_(*weather*) fixed to *p*_*weather*_ = 0.8. Point estimates and standard errors for the coefficients are reported in Table 2, and relative contributions are pictured in Fig 4: rhinovirus explained 6.97% (CI 4.27%–10.28%) of all the IPD cases, 2.48% (CI 0.51%–4.52%) were attributed to RSV, and only 0.67% (CI < 0.01%–1.69%) were attributed to Flu. Overall, the three viruses accounted for 10.12% (CI 7.18%–13.77%) of IPD cases at population level.

**Table 2:** Coefficient estimates for the model of IPD including Flu, rhinovirus, and RSV as covariates.

	Estimate	Std. Error
*α*	−3.1831	0.0066
*γ*	−0.4572	0.0052
*δ*	−0.0838	0.0034
*log*(*ψ*)	3.5064	0.0080
*log*(*τ*)	−1.2946	0.3240
*log*(*θ*)	2.9821	0.0193
*log*(*ζ*)	1.8944	0.0742
*log*(*λ*)	5.8521	2e-04

Abbreviations: IPD, invasive pneumococcal disease; RSV, respiratory syncytial virus; Std., standard

Selected plots displaying age-specific incidence can be found in S4–S6 Figs. For consistency, we used for all age groups the distributional assumption and the endemic component that fitted the univariate time series best (model D). Thus, when considering attribution of IPD to Flu, model selection started by considering the model in Eq 3: *IPD*_*t*,*a*_|*IPD*_*t*−1,*a*_~*NegBin*(*μ*_*IPD*,*t*,*a*_,*ψ*_*a*_) where

$$\begin{array}{c} \mu_{IPD,t,a}=pop_{t,a}[exp(\alpha_{a}+\gamma_{a}\sum_{q=1}^{5}w_{q}(weather)temp_{t-q}+\delta_{a}\sum_{q=1}^{5}w_{q}(weather)rain_{t-q})]+\\ +\tau_{a}FluA_{t-1,a}+\lambda_{a}IPD_{t-1,a}+\phi_{a}\sum_{k\neq a}c_{k,a}IPD_{t-1,k\neq a} \end{array}$$ (5)

However, this required estimating 35 coefficients, not a very parsimonious option. Hence, we tried model reduction by testing whether any of the coefficients could be the same across groups. Full model comparison is reported in S1 Table. AIC decreased from 13,218.85 (model G, with all age-specific coefficients) to 13,216.32 by using a shared rainfall coefficient, i.e., *δ*_*a*_ = *δ* for any age (model H). Finally, the utility of multiple lags for Flu and IPD was considered, but once again a benefit from including past values only pertained to weather variables.

**Fig 5:**
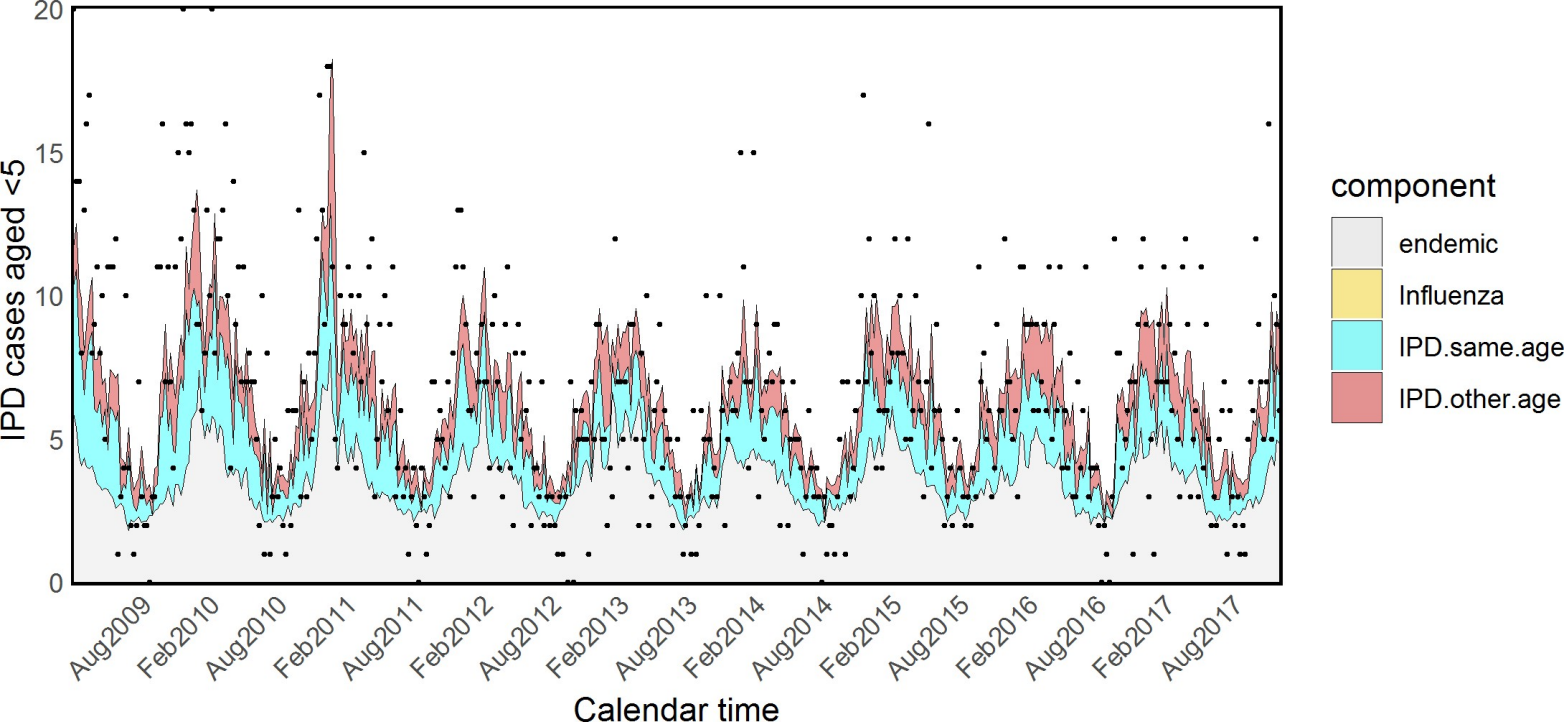
Model I: Fitted IPD values for infants. IPD, invasive pneumococcal disease.

**Fig 6:**
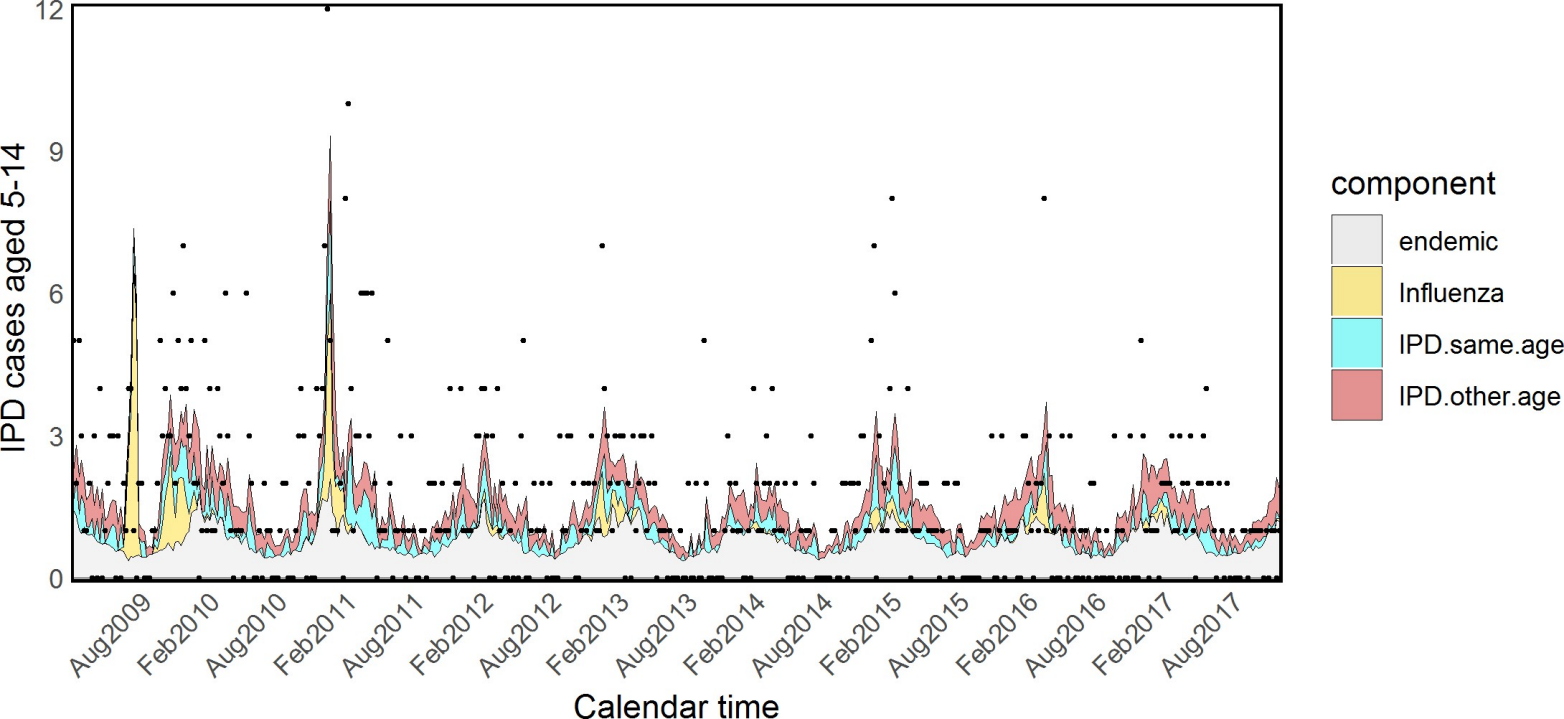
Model I: Fitted IPD values for school-age children. IPD, invasive pneumococcal disease.

**Fig 7:**
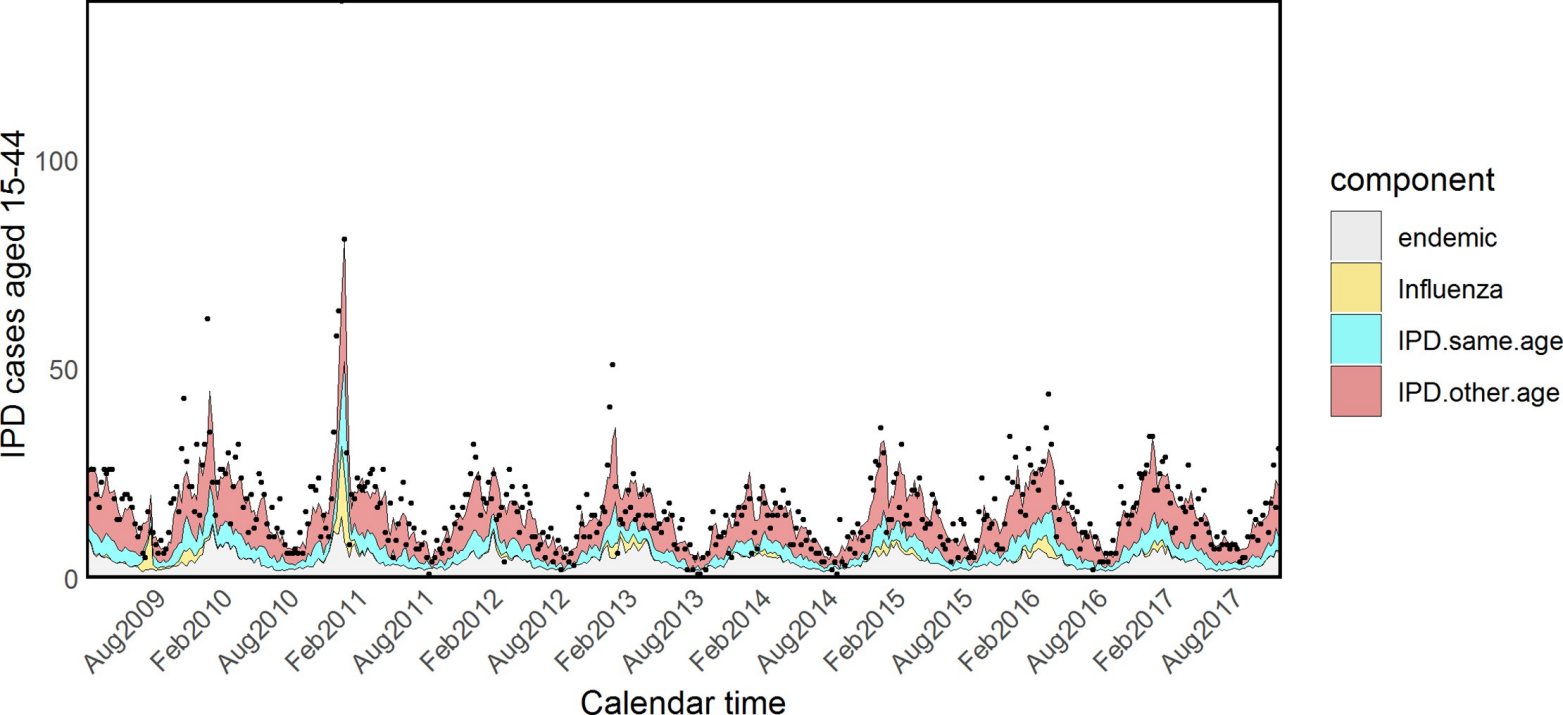
Model I: Fitted IPD values for young adults. IPD, invasive pneumococcal disease.

**Fig 8:**
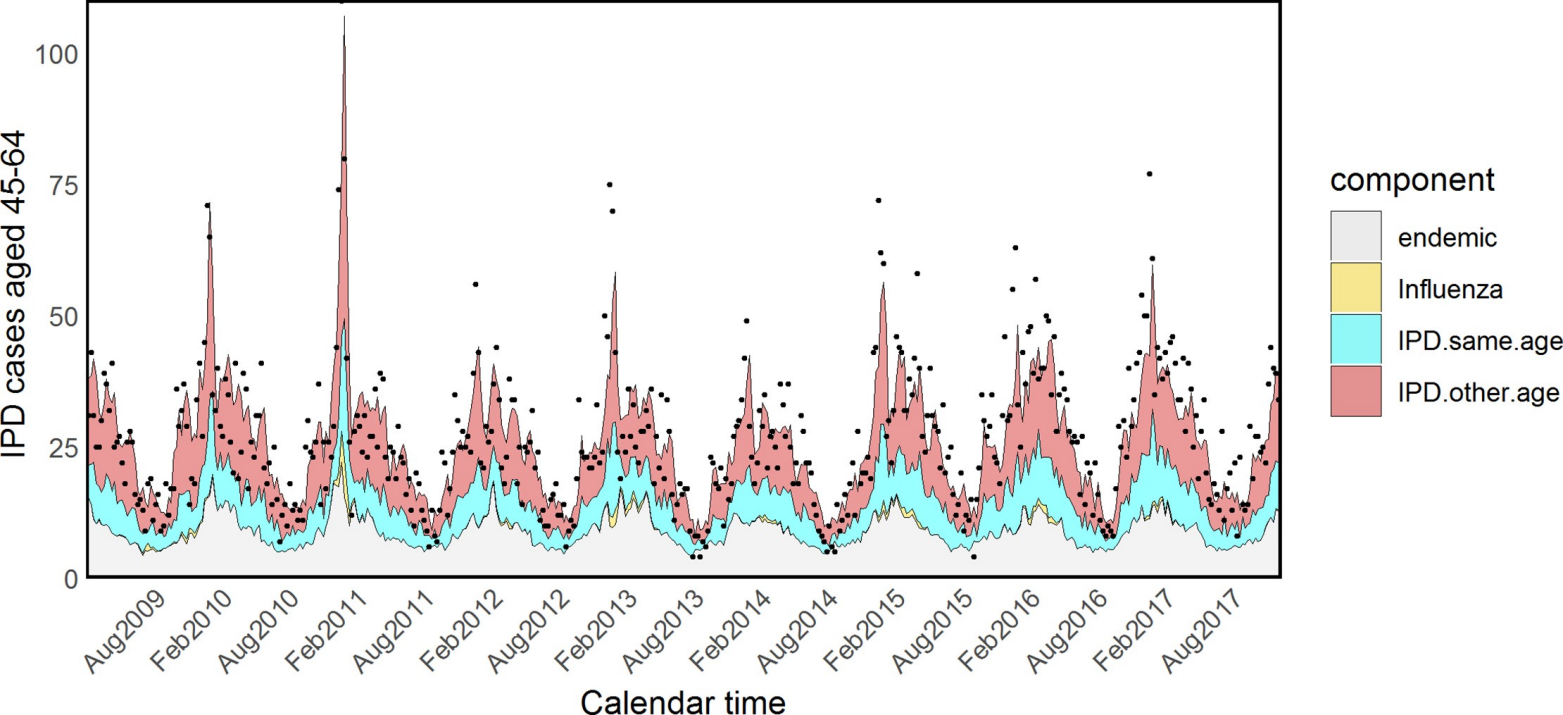
Model I: Fitted IPD values for the 45–64 age group. IPD, invasive pneumococcal disease.

**Fig 9:**
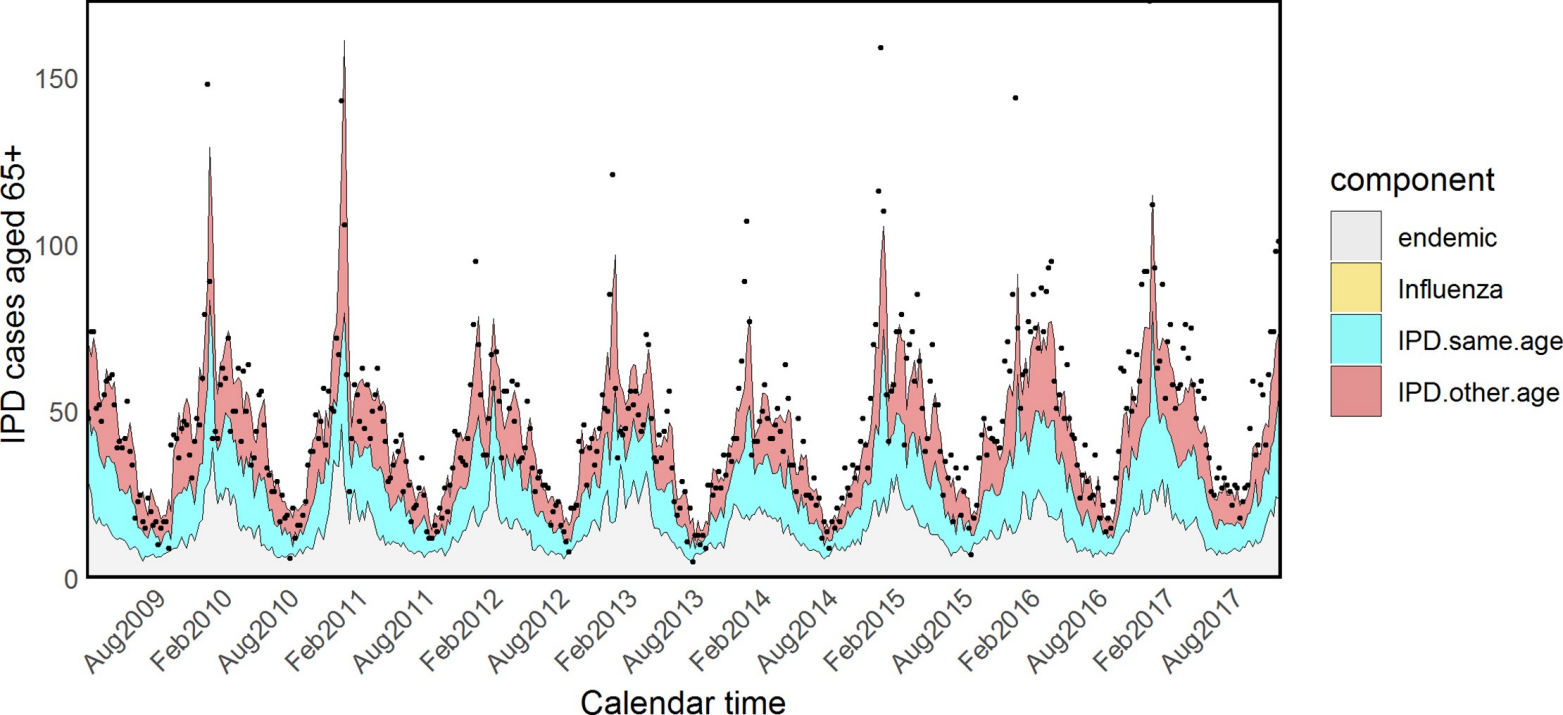
Model I: Fitted IPD values for the elderly. IPD, invasive pneumococcal disease.

Estimated coefficients and standard errors for model H are shown in S2 and S3 Tables. The *τ*_*a*_ parameters associated with influenza were quite heterogeneous across age groups, showing an inverse U–shaped tendency: almost null in young children and the elderly and more prominent in other age groups. However, because of the very small size and associated large uncertainty of the parameters *τ*_5_ and *τ*_65+_, we refitted the model fixing them to zero (model I). The attributed proportions of IPD cases estimated from this model are reported in Table 3, estimated coefficients and standard errors are shown in Tables 4 and 5, and fitted values for all age groups are plotted in Figs 5–9.

**Table 3:** Model I: Relative proportions (%) of IPD cases attributed to pneumococcal transmission within and across age groups and to influenza overall or in the pandemic period.

Age, years	Pneumococcal Transmission		Influenza A	
	Within Group	Across Groups	Overall	AH1N1pmd09
<5	26.42 (16.16–34.49)	18.73 (4.67–33.14)	0.00	0.00
5−14	15.70 (5.32–24.17)	27.32 (2.18–55.97)	8.40 (4.12–13.66)	18.30 (9.43–28.16)
15−44	19.47 (10.33–27.07)	50.67 (38.79–63.16)	3.55 (1.64–5.76)	6.07 (2.83–9.76)
45−64	23.65 (14.79–31.03)	41.64 (31.61–51.49)	0.92 (<0.01–2.94)	1.19 (<0.01–3.78)
65+	33.02 (24.89–39.88)	34.45 (26.02–43.24)	0.00	0.00

Abbreviations: IPD, invasive pneumococcal disease.

**Table 4:** Model I: Coefficient estimates for the age-specific model of IPD including Flu.

Age, years	*α*	*γ*	*δ*	*log*(*ψ*)	*log*(*τ*)	*log*(*λ*)	*log*(*ϕ*)
<5	−2.2818	−0.3173	−0.0372	2.5131	-	2.1816	1.2908
5–14	−4.4221	−0.3688	−0.0372	1.5905	−3.2617	2.2835	1.3644
15–44	−4.0347	−0.4770	−0.0372	3.024	−1.6285	3.7285	4.0934
45–64	−2.7756	−0.3419	−0.0372	3.1729	−2.1502	3.4786	3.6104
65+	−1.9382	−0.4640	−0.0372	3.1881	-	3.4232	4.2462

Abbreviation: IPD, invasive pneumococcal disease.

**Table 5:** Model I: Coefficient standard errors for the age-specific model of IPD including Flu.

Age, years	*α*	*γ*	*δ*	*log*(*ψ*)	*log*(*τ*)	*log*(*λ*)	*log*(*ϕ*)
<5	0.0033	0.0017	0.0012	0.0385	-	0.0109	0.0142
5−14	0.0145	0.0092	0.0012	0.0718	0.1035	0.0517	0.0222
15−44	0.0063	0.006	0.0012	0.0220	0.0661	0.0068	9e-04
45−64	0.0128	0.0113	0.0012	0.0158	0.7316	0.0036	0.001
65+	0.0017	0.0011	0.0012	0.0101	-	0.0018	0.0012

Abbreviation: IPD, invasive pneumococcal disease.

According to model I, IPD was driven by Flu in school-age children (8.40%, CI 4.12%–13.66%) and adults aged 15–44 years (3.55%, CI 1.64%–5.76%), and these components were strikingly higher in the pandemic period: 18.30% (CI 9.43%–28.16%) and 6.07% (CI 2.83%–9.76%), respectively.

Adding rhinovirus in the best-fitting model I led to the biggest AIC reduction, from 13,216.32 to 13,167.31, when its contribution was quantified by an age-specific coefficient *θ*. Lastly, the addition of RSV further contributed to AIC reduction (13,153.89). Hence, the final model took the form

$$\begin{array}{c} \mu_{IPD,t,a}=pop_{t}\nu_{t,a}+\tau_{a}Flu_{t-1}+\theta_{a}rhinovirus_{t-1}+\zeta_{a}RSV_{t-1}++\\ +\lambda_{a}IPD_{t-1}+\phi_{a}\sum_{k\neq a}c_{j,i}IPD_{k\neq a,t-1} \end{array}$$ (6)

where $\nu_{t,a}=exp\left[\alpha_{a}+\gamma_{a}\sum_{q=1}^{5}w_{q}(weather)temp_{t-q}+\delta \sum_{q=1}^{5}w_{q}(weather)rain_{t-q})\right]$. As for the model with only Flu, because of large uncertainty about coefficients close to 0, the coefficients *θ*_5−14_, *θ*_15−44_, *ζ*_5−14_, and *ζ*_15−44_ were fixed to zero (models J and K). Fitted values for all age groups are plotted in S7–S11 Figs; coefficients and standard errors are listed in S4 and S5 Tables, whereas the relative contribution of the components is described in Table 6.

**Table 6:** Model K: Relative proportions (%) of IPD cases attributed to pneumococcal transmission within and across age groups, to influenza, rhinovirus, and RSV.

Age	Endemic	Influenza	Rhinovirus	RSV	IPD Same Age	IPD Other Age
<5	50.35 (34.23–66.91)	0.00	4.49 (<0.01–12.20)	1.31 (<0.01–5.26)	26.32 (16.24–33.95)	17.53 (3.28–32.61)
5–14	49.94 (21.31–75.08)	8.54 (4.21–13.43)	0.00	0.00	15.68 (5.06–24.10)	25.84 (1.65–55.97)
15–44	26.35 (15.85–38.35)	3.56 (1.69–5.82)	0.00	0.00	19.40 (10.59–26.88)	50.70 (38.19–63.20)
45–64	29.24 (20.87–39.76)	0.91 (<0.01–2.83)	5.43 (2.23–8.91)	4.18 (1.58–6.91)	17.15 (8.04–24.27)	43.09 (32.64–53.07)
65+	29.05 (21.65–38.27)	0.00	5.68 (3.03–8.32)	3.91 (1.83–6.38)	23.75 (14.97–30.68)	37.62 (29.41–46.45)

Abbreviations: IPD, invasive pneumococcal disease; RSV, respiratory syncytial virus.

Model K showed that the association between RSV and IPD was strongest in the elderly (3.91%, CI 1.83%–6.38%, of cases in the 65+ group and 4.18%, CI 1.58%–6.91% of cases in the 45–64 group), and rhinovirus played an important role in the same age groups: 5.43% (CI 2.23%–8.91%) in the 45–64 group and 5.68% (CI 3.03%-8.32%) in the 65+ group.

## Discussion

Using English surveillance data, we quantified the magnitude of the interaction between influenza virus and *S*. *pnuemoniae* in seasonal and pandemic settings by proposing a multivariate extension of the HHH modelling framework. Such interaction was estimated to be quite small when looking at population-wide counts (model D). These results are consistent with previous research, showing a small association at aggregate level [24]. We found evidence to support the hypothesis of an age-specific interaction [34], the contribution of Flu towards IPD being significant in school-age children and adults aged 15–44 but not in other age groups (model I). Moreover, the components of IPD explained by influenza were strikingly higher during the 2009 pandemic period in the same age groups. This supports findings of Weinberger and colleagues [49]. Other viruses also appeared to interact with *S*. *pneumoniae* with various intensities across age groups: both RSV and rhinovirus played an important role in 45- to 64- and 65+-year-olds (models F and K respectively). Such findings support previous evidence of interplay among these pathogens, with differential behaviour across ages [50, 51].

The additive structure of the model allowed us to quantify the contribution of multiple viruses to the IPD counts, and at the same time the multivariate age-specific model allowed a better characterisation of each of these interactions. Another important advantage of the modelling framework used here was the potential to assess pneumococcal disease transmission. Our findings suggested that 50.70% (CI 38.19%–63.20%) of pneumococcal disease in adults aged 15–44 years, potential parents of young children, was transmitted from other age groups. Transmission within group, on the other hand, prevailed in preschool children and 65+-year-olds: 26.32% (CI 16.24%–33.95%) and 23.75% (CI 14.97%–30.68%), respectively (model K). We speculate this could be due to higher incidence of IPD in care homes or in immunocompromised people.

Finally, the endemic component captured considerable proportions of IPD incidence in all age groups. We can think of this seasonal background as the proportion of disease probably due to some common environmental factors. The adequacy of temperature and rainfall observations to replace harmonic functions, supported by enhanced model fit both at aggregate and age-specific level, reinforces this hypothesis. The appropriateness of shared coefficients for rainfall also suggests that disease seasonality has similar timing across the entire population.

Any estimates of association between two pathogens such as influenza and pneumococcus, both transmitted through air droplets and typical of the winter season, are fraught with uncertainties. First, the validity of this modelling strategy relies on the assumption that viral surveillance is consistent over time and adequately represents the true burden in the population [52]. If this assumption does not hold, apparent trends over time might be due to improved diagnostics or enhanced reporting rather than to a real change in incidence. Our analysis accounted for imperfect detection and reporting of influenza using primary care data and integrating results of virological testing. Reverse transcription PCR (RT-PCR) testing of respiratory specimens is the “gold standard” for confirming a viral presence, and the long-term sentinel scheme implies doctors are not solicited to test because of increased alertness. However, only a subset of infected people seek healthcare, symptomatic disease being a necessary condition. Further, test results may still be subject to misclassification depending on when the specimen is obtained during the illness, and timeliness in reporting results can vary across clinical practices. Future work might exploit the availability of serological testing to better approximate the magnitude and timing of any influenza outbreak. Finally, we simply multiplied the proportion of positive samples by the ILI rates, whereas a joint modelling approach would take uncertainty into account. In terms of IPD data, we believe that testing policies must be consistent over time because of the life-threatening nature of such a condition. Thanks to UK-wide guidelines [40], we believe that reporting was relatively stable over time, making surveillance data as reliable as possible. Nonetheless, the limited numbers of cases, especially in the age-specific analysis, made the resulting estimates uncertain.

Despite our efforts to mimic disease mechanisms, a number of assumptions were made in our analysis. First, we assumed the lag between events to be at least 1 week. Dealing with weekly data, this was the best approximation we could choose within a biologically plausible range [53]. However, the infectious time might be shorter than the chosen time unit, and any unknown delay in reporting might introduce some bias. Second, we assumed autoregressive coefficients to be fixed over time. This implies that pneumococcal transmission has no seasonal behaviour, and likewise, the interaction with influenza is the same in summer and winter. Such an assumption is meant to include any season-specific variation into the endemic component that summarises a number of unknown aspects such as, for example, climatic influence on disease susceptibility. Fixed autoregressive coefficients also helped to keep our model easy to interpret and to avoid overfitting. Third, contact patterns across age groups were approximated by the POLYMOD matrix, which was estimated on a sample of people living in England in 2005–2006 [48]. Current patterns might be different, and real contact probabilities might not be constant over time. Nevertheless, the use of age-structured contact patterns led to improved model fit compared to an assumption of random mixing between age groups. Fourth, we are aware that pneumococcus is often carried by healthy individuals who might silently transmit the pathogen. In the present analysis, we could not disentangle pneumococcal carriage from disease, as that would have required detailed individual information, such as testing asymptomatic people to detect carriage. Compartmental models with mechanistic assumptions could be employed in future work to fully reconstruct the epidemic process [54].

Despite the above limitations, our modelling strategy successfully improved existing understanding of interaction between multiple pathogens: our estimates are valuable to quantify the possible contribution of influenza to the burden of IPD in a future pandemic of influenza with similar characteristics to the 2009 pandemic, bearing in mind that it was considered relatively mild, compared for example to the 1918 pandemic. The proposed model could be usefully employed by many countries that rely on infectious disease surveillance for informing policy, in terms of both pandemic preparedness and pneumococcal vaccine introduction. Furthermore, we believe our approach could be valuably applied to retrospectively investigate relationships of other notifiable diseases. For example, the contribution of viruses to secondary bacterial infections due to *Staphylococcus aureus* and *Streptococcus pyogenes* requires future investigation to better inform antibiotic prescription policies.

We have clarified the role of the influenza virus on severe pneumococcal infections, in both seasonal and pandemic settings. Although the seasonal contribution does not appear to be relevant, the interaction with pandemic strains resulted significant, particularly in younger age groups. These findings have implications for pandemic preparedness in terms of advising on antibiotic stockpiles, for which currently there is no clear evidence. Finally, a further extension could tackle spatial dynamics if region-specific counts are available, as they would provide a more detailed understanding of spatiotemporal dependencies inherent to the disease and its drivers. However, dynamics of diseases involve processes at different scales of hosts, space, and time, and the attribution of a causal role of one pathogen or another remains a challenging problem [55].

## Supporting information

S1 Checklist GATHER checklist. (PDF)

S1 Appendix Analysis strategy. (PDF)

S1 Fig Fitted IPD values all ages. IPD, invasive pneumococcal disease.
(TIFF)

S2 Fig RSV incidence rate. RSV, respiratory syncytial virus.
(TIFF)

S3 Fig Rhinovirus incidence rate. (TIFF)

S4 Fig Observed counts: 15–44 years old. (TIFF)

S5 Fig Observed counts: 45–64 years old. (TIFF)

S6 Fig Observed counts: 65+ years old. (TIFF)

S7 Fig Model K: Fitted IPD values for infants. IPD, invasive pneumococcal disease.
(TIFF)

S8 Fig Model K: Fitted IPD values for school-age children. IPD, invasive pneumococcal disease.
(TIFF)

S9 Fig Model K: Fitted IPD values for young adults. IPD, invasive pneumococcal disease.
(TIFF)

S10 Fig Model K: Fitted IPD values for the 45–64 age group. IPD, invasive pneumococcal disease.
(TIFF)

S11 Fig Model K: Fitted IPD values for the elderly. IPD, invasive pneumococcal disease.
(TIFF)

S1 Table Multivariate model comparison in terms of AIC and one-step-ahead forecast (log[s(P,x)]). AIC, Akaike information criterion.
(PDF)

S2 Table Model I: Coefficient estimates for the age-specific model of IPD including Flu. Since Flu coefficients *τ*_<5_ and *τ*_65+_ were very small, we refitted the model fixing them to 0 to make sure the other parameter estimates were not sensitive to such an assumption. IPD, invasive pneumococcal disease.
(PDF)

S3 Table Model I: Standard error estimates for the age-specific model of IPD including Flu. Uncertainty around coefficients *τ*_<5_ and *τ*_65+_ was not well estimated. IPD, invasive pneumococcal disease.
(PDF)

S4 Table Model K: Coefficient estimates for the age-specific model of IPD including Flu, rhinovirus, and RSV. IPD, invasive pneumococcal disease; RSV, respiratory syncytial virus. (PDF)

S5 Table Model K: Coefficient standard errors for the age-specific model of IPD including Flu, rhinovirus, and RSV. IPD, invasive pneumococcal disease; RSV, respiratory syncytial virus.
(PDF)

## Abbreviations

AIC: Akaike information criterion
ARI: acute respiratory illness
ARIMA: autoregressive integrated moving average
CI: confidence interval
GATHER: Guidelines for Accurate and Transparent Health Estimates Reporting
GLM: generalized linear model
hMPV: human metapneumovirus
ILI: influenza-like illness
IPD: invasive pneumococcal disease
LRTI: lower respiratory tract infection
PHE: Public Health England
RCGP RSC: Royal College of General Practitioners Research and Surveillance Centre
RT-PCR: reverse transcription PCR
RSV: respiratory syncytial virus
SGSS: Second Generation Surveillance System
