# Supplementary material for: Estimating age-stratified influenza-associated invasive pneumococcal disease in England: A time-series model based on population surveillance data
Source: PLoS Med. 2019 Jun 27;16(6):e1002829. doi: 10.1371/journal.pmed.1002829 (PMC6597037; doi:10.1371/journal.pmed.1002829)
Supplement: S1 Appendix — (PDF) [file pmed.1002829.s002.pdf]

Analysis strategy

We started by considering the time series referring to the entire population, without age stratification, and we assessed which formulation of model (1) fitted our data best in terms of AIC over all the study period. We first tested the usefulness of an overdispersion parameter by comparing distributional assumptions (Poisson vs negative binomial). Then, considering the mean is decomposed as $\mu_t = pop_t\nu_t + \lambda Y_{t-1} + \tau X_{t-1}$, we compared different expressions for the endemic component ν_t. Yearly seasonality for weekly data is described through the general formulation

$$log(\nu_t) = \alpha + \sum_{s=1}^{S} \gamma_s sin\Big(\frac{2\pi st}{52}\Big) + \delta_s cos\Big(\frac{2\pi st}{52}\Big) \tag{1}$$

where α is an intercept and γ_s and δ_s quantify the amplitude of the sine-cosine waves. We first assessed the optimal number S of harmonic functions to be included, and in a second stage we checked whether replacing the sine-cosine waves with rainfall and temperature information better characterised differences across winters. Finally, we extended the model to include multiple lags for covariates, relaxing the assumption of temporal dependence limited to one week, as suggested by [1]. Denoting by Q the number of lags considered, the mean was written as

$$\mu_t = pop_t\nu_t + \lambda \sum_{q=1}^{Q} w_q(y) Y_{t-q} + \tau \sum_{q=1}^{Q} w_q(x) X_{t-q} \tag{2}$$

where $w_q(y)$ and $w_q(x)$ are normalized lag weights defined according to a geometric structure, with parameters p_x and p_y shaping the respective exponential decays, i.e.

$$w_q(y) = \frac{p_y(1-p_y)^{q-1}}{\sum_{q=1}^{Q} p_y(1-p_y)^{q-1}} \tag{3}$$

To avoid overfitting, we compared the performance of our different model formulations in terms of one-step-ahead forecasts. Such performance is usually measured in terms of scoring rules, where a score is intended as a penalty $s(P, x)$, that is a function of the observed counts x and predictive distribution P. When dealing with count data, the logarithmic score $log(s(P, x)) = -logP_x$ is a standard choice: it is simply a log transformation of the probability mass P at the observed count x [2,3]. Hence, for each choice of lags and endemic variables, we computed the logarithmic scores for a set of forecasts and we took their mean as a summary measure.

The same model assessment procedure was replicated when looking at age-specific counts: the model in equation (3) was simultaneously fitted to the five age groups, initially assuming that all the coefficients could be age-specific. We first performed model selection using AIC to evaluate whether some coefficients could be shared across age groups: model fit was assessed in a sequential way, testing at each stage which of the components led to a larger AIC reduction when associated with non age-specific coefficients.

References

1. Johannes Bracher and Leonhard Held. Periodically stationary multivariate autoregressive models. *arXiv preprint arXiv*,1707.04635, 2017.

2. Claudia Czado, Tilmann Gneiting, and Leonhard Held. Predictive model assessment for count data. *Biometrics*, 65(4):1254–1261,2009.

3. Leonhard Held, Sebastian Meyer, and Johannes Bracher. Probabilistic forecasting in infectious disease epidemiology: the 13th Armitage lecture. *Statistics in medicine*, 36(22):3443–3460,2017.
