# Supplementary figures and images for: Estimating age-stratified influenza-associated invasive pneumococcal disease in England: A time-series model based on population surveillance data
Source: PLoS Med. 2019 Jun 27;16(6):e1002829. doi: 10.1371/journal.pmed.1002829 (PMC6597037; doi:10.1371/journal.pmed.1002829)

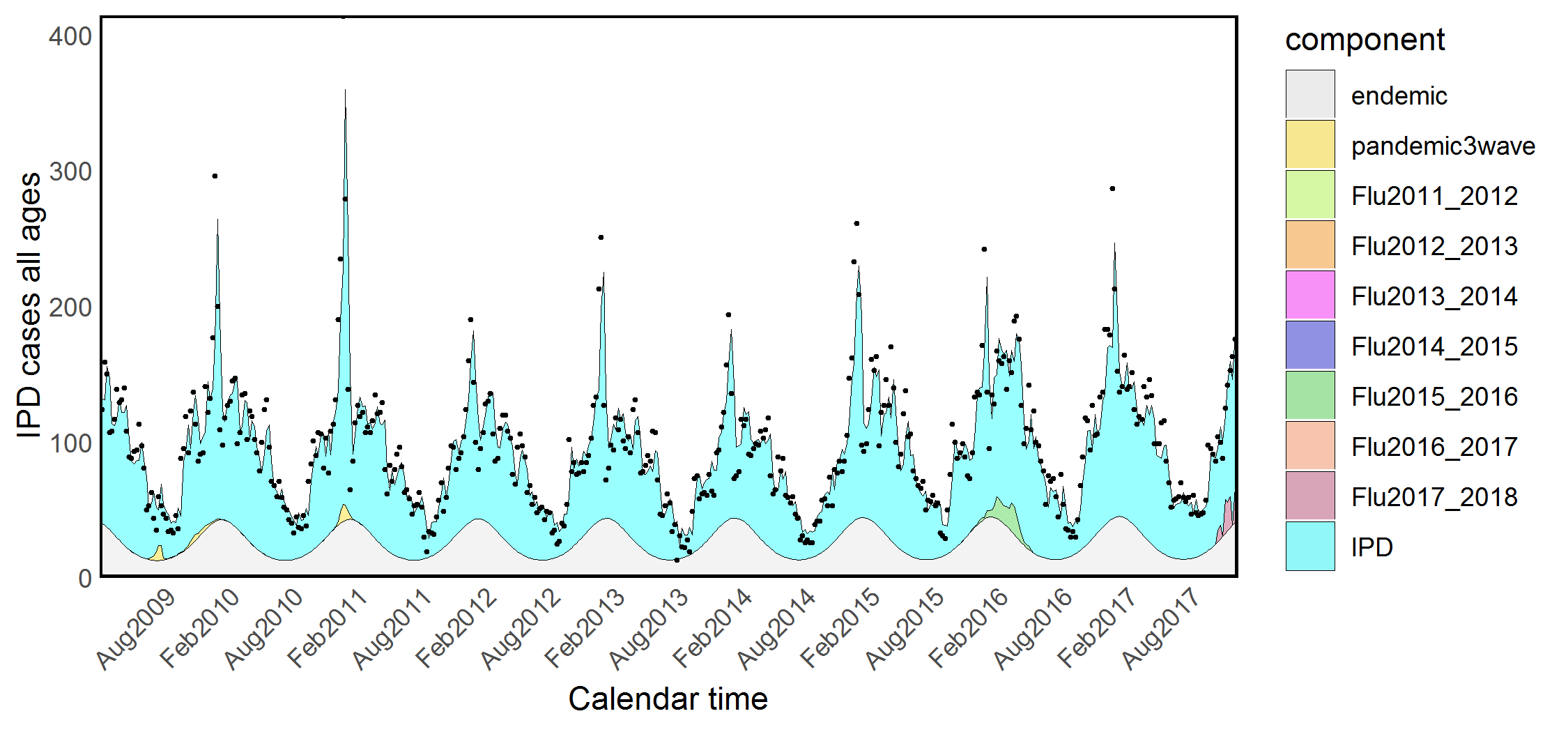

Supplement: S1 Fig — IPD, invasive pneumococcal disease. (TIFF) [file pmed.1002829.s003.tiff]

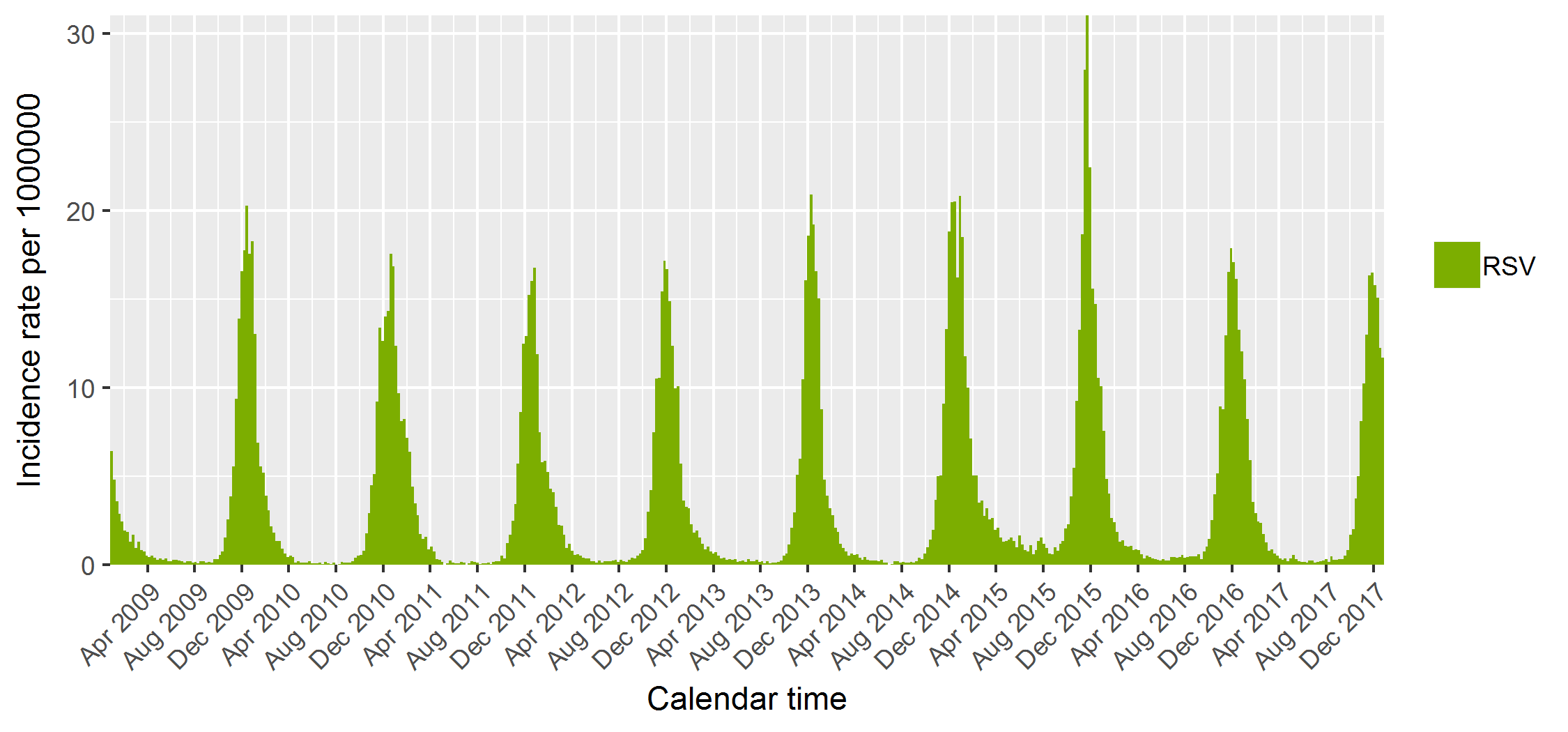

Supplement: S2 Fig — RSV, respiratory syncytial virus. (TIFF) [file pmed.1002829.s004.tiff]

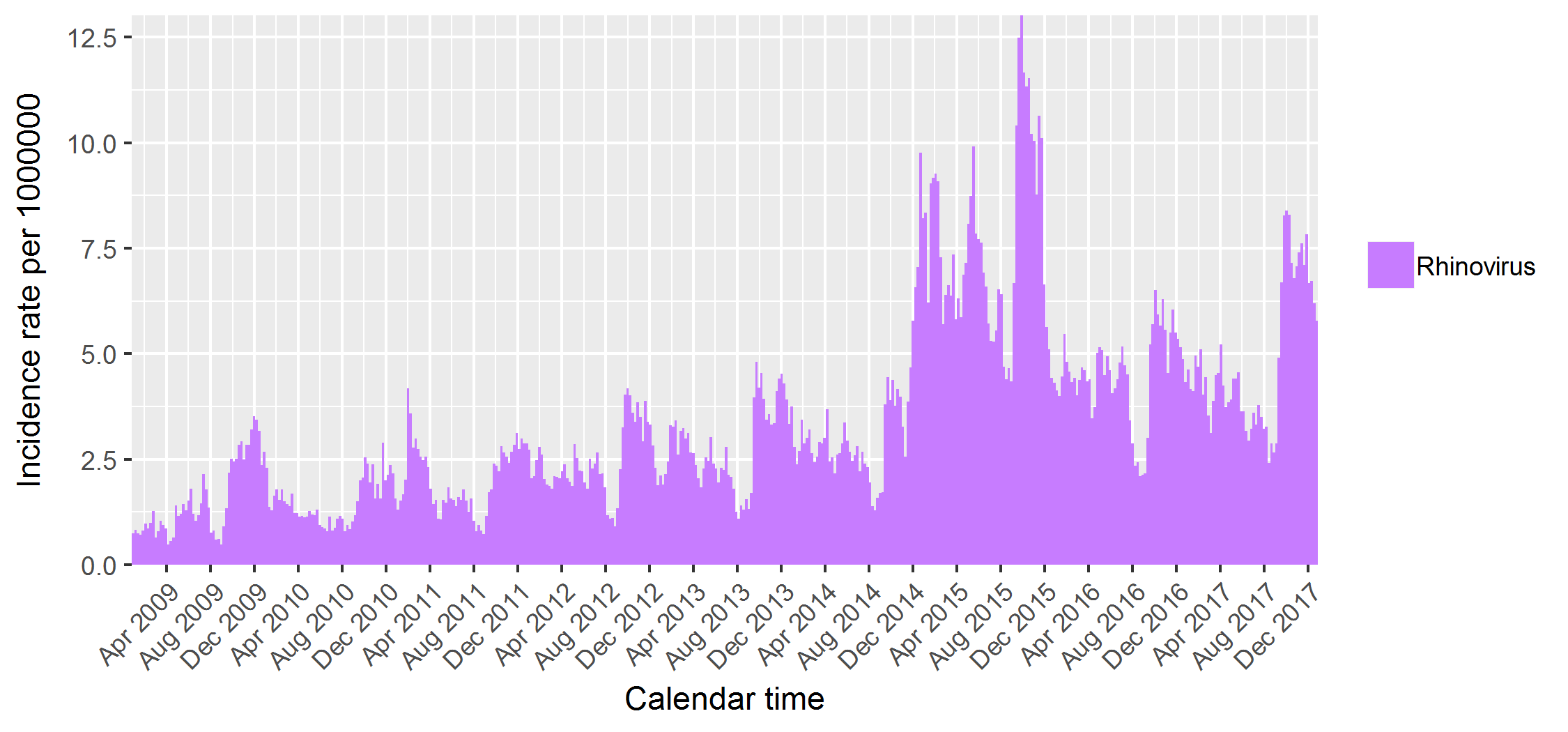

Supplement: S3 Fig — (TIFF) [file pmed.1002829.s005.tiff]

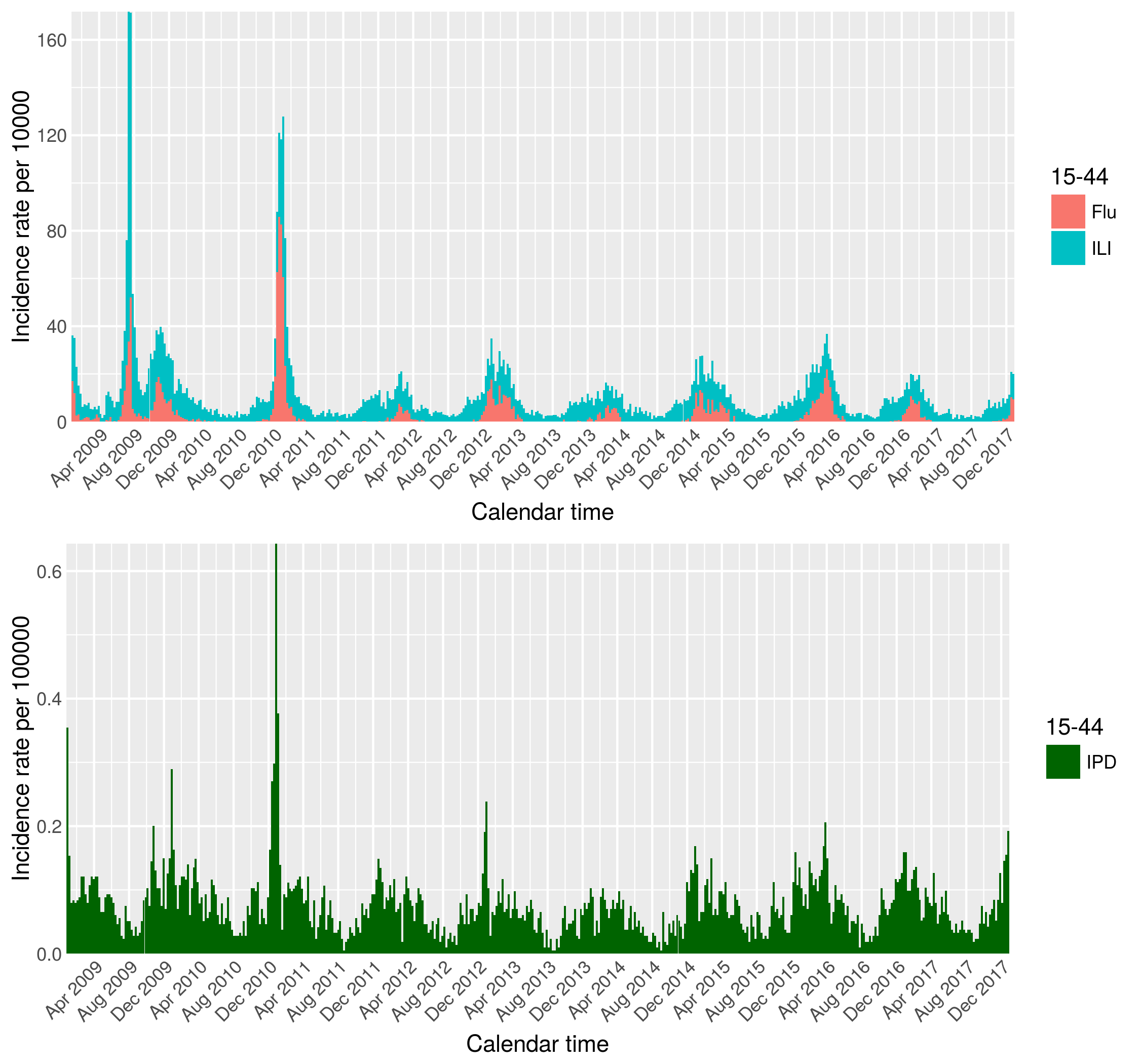

Supplement: S4 Fig — (TIFF) [file pmed.1002829.s006.tiff]

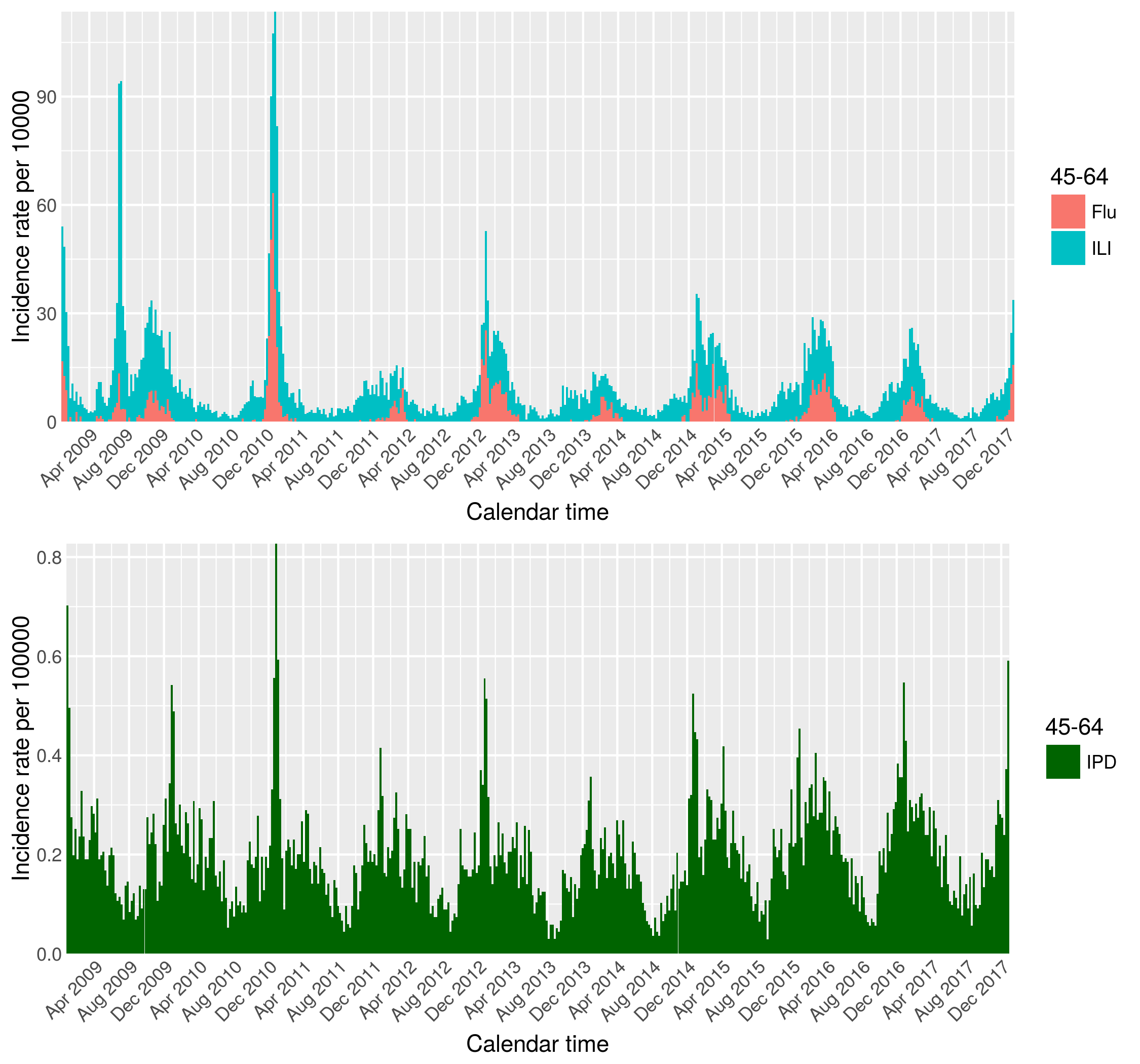

Supplement: S5 Fig — (TIFF) [file pmed.1002829.s007.tiff]

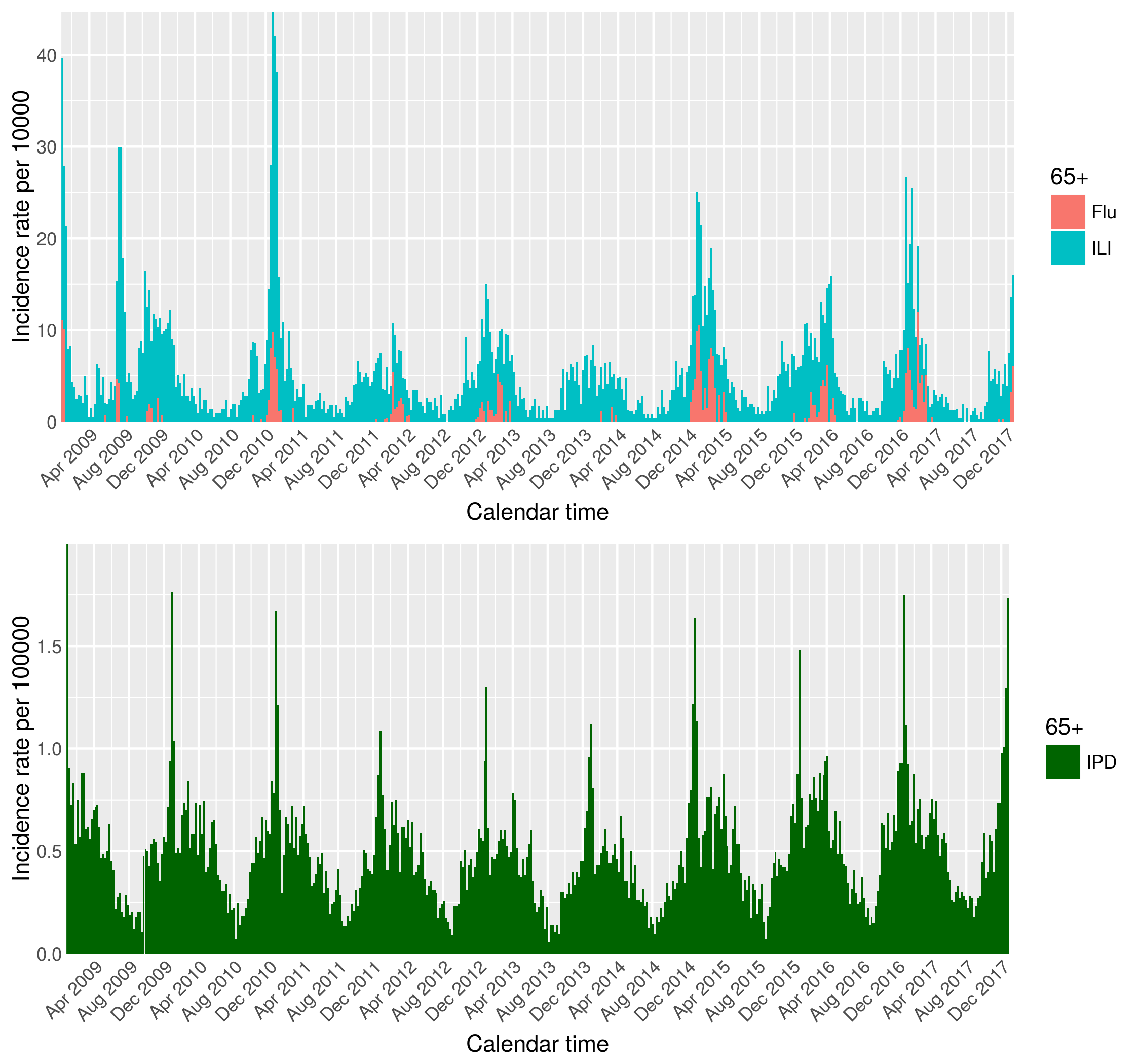

Supplement: S6 Fig — (TIFF) [file pmed.1002829.s008.tiff]

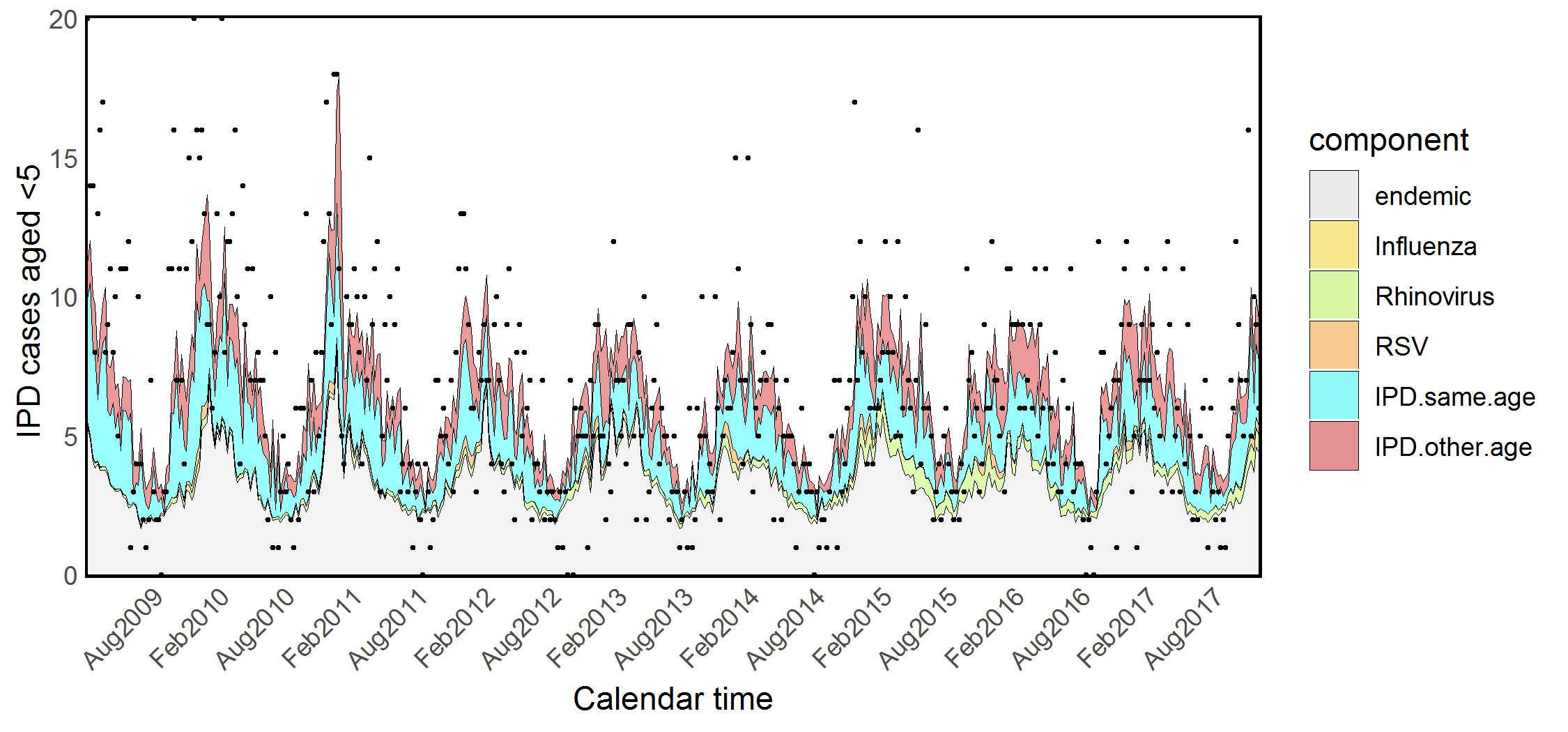

Supplement: S7 Fig — IPD, invasive pneumococcal disease. (TIFF) [file pmed.1002829.s009.tiff]

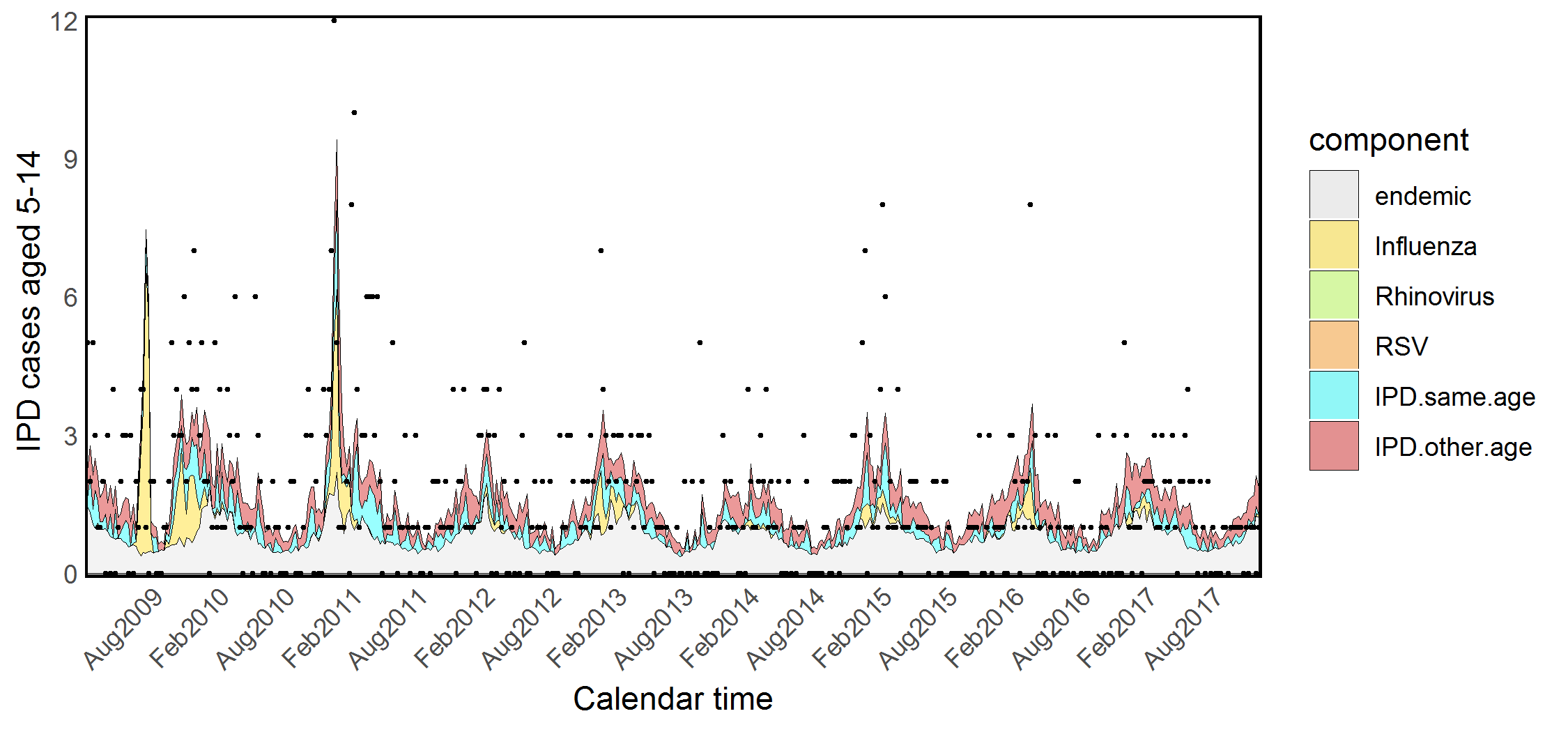

Supplement: S8 Fig — IPD, invasive pneumococcal disease. (TIFF) [file pmed.1002829.s010.tiff]

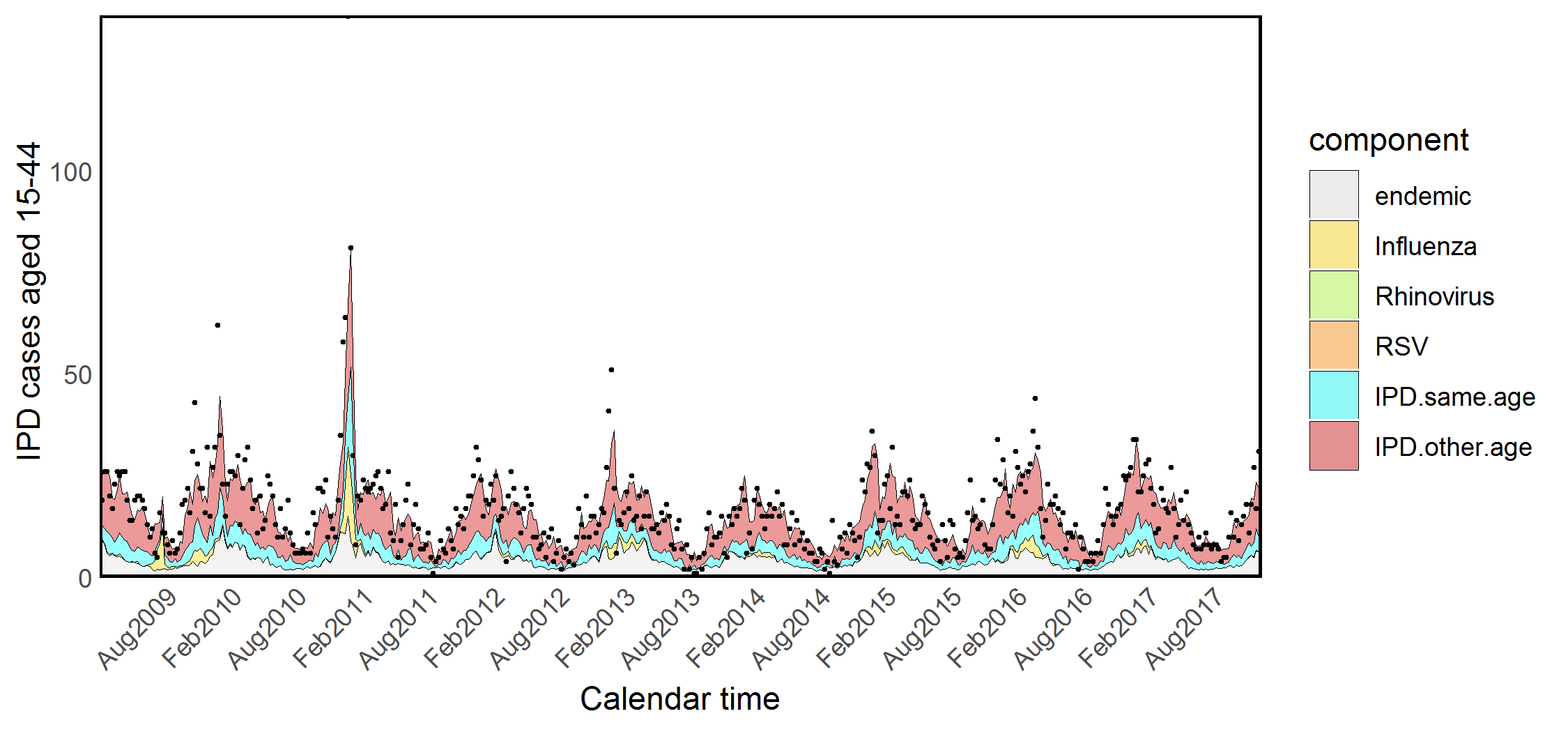

Supplement: S9 Fig — IPD, invasive pneumococcal disease. (TIFF) [file pmed.1002829.s011.tiff]

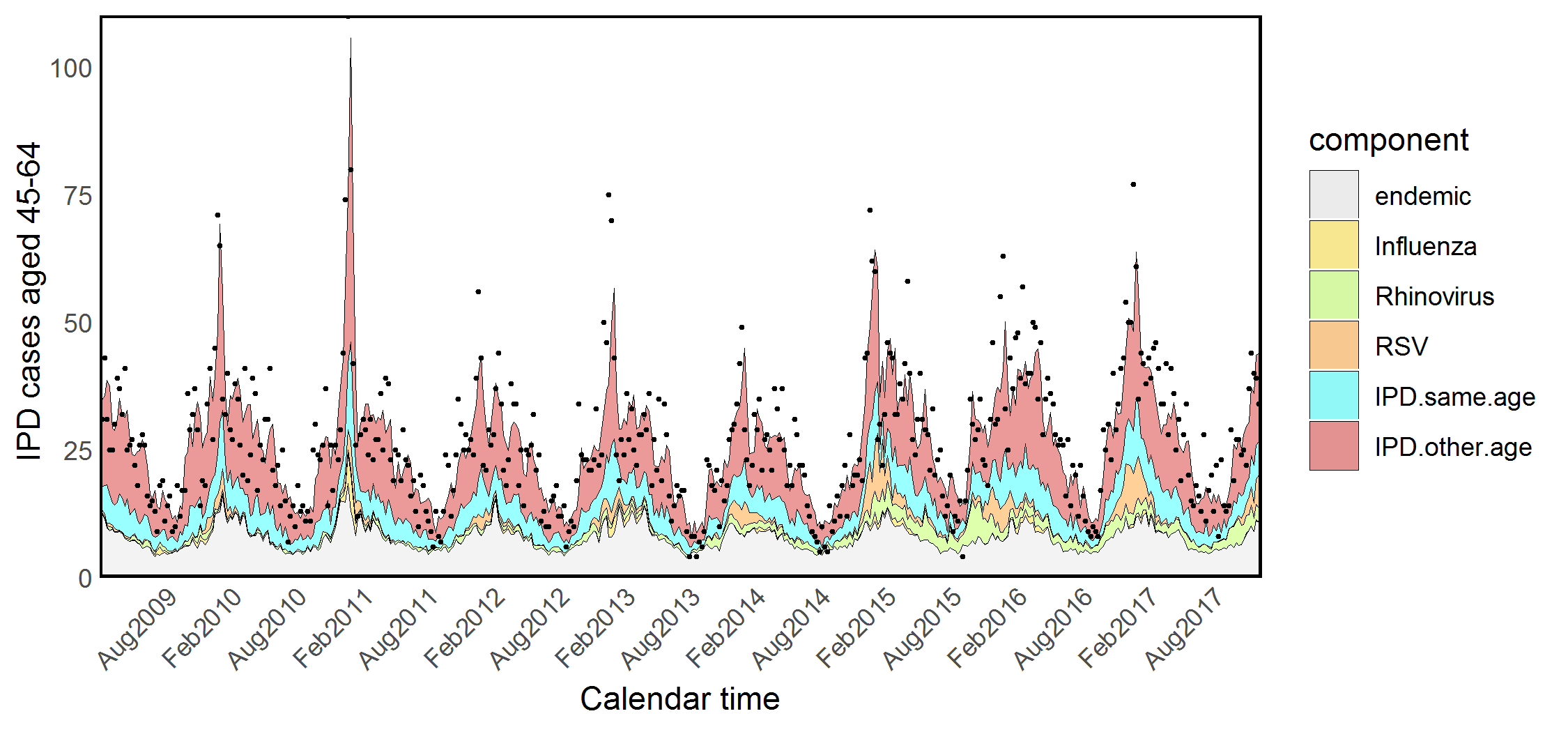

Supplement: S10 Fig — IPD, invasive pneumococcal disease. (TIFF) [file pmed.1002829.s012.tiff]

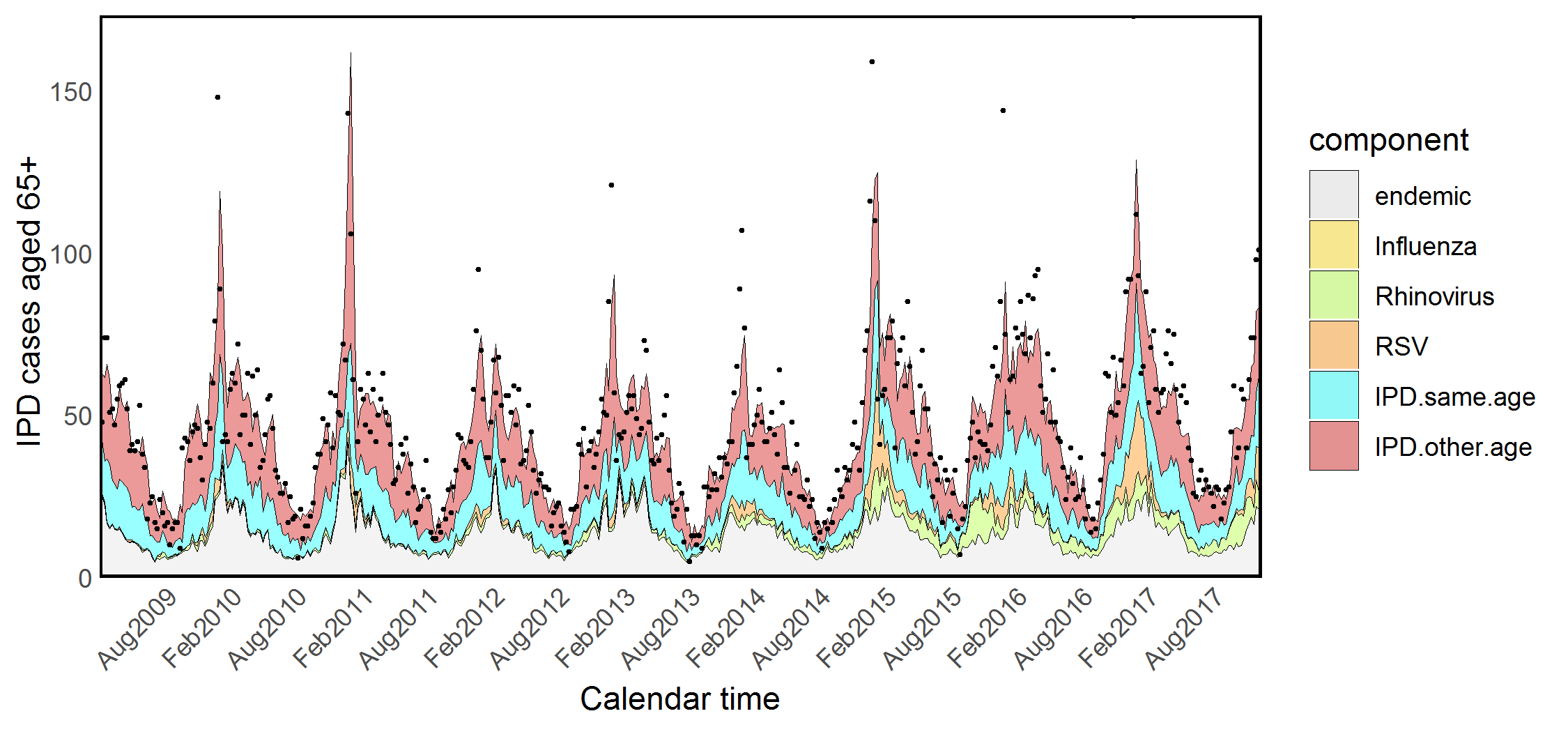

Supplement: S11 Fig — IPD, invasive pneumococcal disease. (TIFF) [file pmed.1002829.s013.tiff]
