# Supplementary material for: Estimating age-stratified influenza-associated invasive pneumococcal disease in England: A time-series model based on population surveillance data
Source: PLoS Med. 2019 Jun 27;16(6):e1002829. doi: 10.1371/journal.pmed.1002829 (PMC6597037; doi:10.1371/journal.pmed.1002829)
Supplement: S1 Table — AIC, Akaike information criterion. (PDF) [file pmed.1002829.s014.pdf]

	covar	shared pars	AIC
G	Flu	all age-spec	13218.85
H	Flu	$\delta_a = \delta$	13216.32
I	Flu	$\delta_a = \delta$, $\tau_{<5} = \tau_{65+} = 0$	13212.32
J	Flu+rhinov	$\delta_a = \delta$, $\tau_{<5} = \tau_{65+} = \theta_{5-14} = \theta_{15-44} = 0$	13160.70
K	Flu+rhinov+RSV	$\delta_a = \delta$, $\tau_{<5} = \tau_{65+} = \theta_{5-14} = \theta_{15-44} =$ $= \zeta_{5-14} = \zeta_{15-44} = 0$	13143.67

S1 Table . Multivariate model comparison in terms of AIC and one-step ahead forecast (log(s(P,x)))
