# Supplementary material for: Estimating age-stratified influenza-associated invasive pneumococcal disease in England: A time-series model based on population surveillance data
Source: PLoS Med. 2019 Jun 27;16(6):e1002829. doi: 10.1371/journal.pmed.1002829 (PMC6597037; doi:10.1371/journal.pmed.1002829)
Supplement: S2 Table — Since Flu coefficients τ<5 and τ65+ were very small, we refitted the model fixing them to 0 to make sure the other parameter estimates were not sensitive to such an assumption. IPD, invasive pneumococcal disease. (PDF) [file pmed.1002829.s015.pdf]

Age	α	γ	δ	$log(\psi)$	$log(\tau)$	$log(\lambda)$	$log(\phi)$
<5	-2.282	-0.317	-0.037	2.513	-13.847	2.182	1.291
$5-14$	-4.422	-0.369	-0.037	1.590	-3.262	2.283	1.364
$15-44$	-4.035	-0.477	-0.037	3.024	-1.629	3.729	4.093
$45-64$	-2.776	-0.342	-0.037	3.173	-2.150	3.479	3.610
65+	-1.938	-0.464	-0.037	3.188	-6.714	3.423	4.246

S2 Table . Model I: Coefficient estimates for the age-specific model of IPD including Flu. Since Flu coefficients $\tau_{<5}$ and τ_{65+} were very small, we refitted the model fixing them to 0, to make sure the other parameter estimates were not sensitive to such an assumption.
