# Supplementary material for: Estimating age-stratified influenza-associated invasive pneumococcal disease in England: A time-series model based on population surveillance data
Source: PLoS Med. 2019 Jun 27;16(6):e1002829. doi: 10.1371/journal.pmed.1002829 (PMC6597037; doi:10.1371/journal.pmed.1002829)
Supplement: S3 Table — Uncertainty around coefficients τ<5 and τ65+ was not well estimated. IPD, invasive pneumococcal disease. (PDF) [file pmed.1002829.s016.pdf]

Age	α	γ	δ	$log(\psi)$	$log(\tau)$	$log(\lambda)$	$log(\phi)$
<5	0.126	0.040	0.024	0.197	177.157	0.179	0.398
5 − 14	0.254	0.086	0.024	0.269	0.354	0.301	0.482
15 − 44	0.182	0.063	0.024	0.148	0.307	0.264	0.127
45 − 64	0.119	0.039	0.024	0.126	1.048	0.221	0.141
65+	0.111	0.034	0.024	0.101	-	0.158	0.147

S3 Table . Model I: Standard error estimates for the age-specific model of IPD including Flu. Uncertainty around coefficients $\tau_{<5}$ and τ_{65+} was not well estimated.
