# Supplementary material for: Estimating age-stratified influenza-associated invasive pneumococcal disease in England: A time-series model based on population surveillance data
Source: PLoS Med. 2019 Jun 27;16(6):e1002829. doi: 10.1371/journal.pmed.1002829 (PMC6597037; doi:10.1371/journal.pmed.1002829)
Supplement: S4 Table — (PDF) [file pmed.1002829.s017.pdf]

Age	α	γ	δ	$log(\psi)$	$log(\tau)$	$log(\theta)$	$log(\zeta)$	$log(\lambda)$	$log(\phi)$
<5	-2.369	-0.319	-0.062	2.524	-	-2.307	-4.000	2.178	1.225
5-14	-4.395	-0.367	-0.062	1.598	-3.245	-	-	2.282	1.308
15-44	-4.034	-0.477	-0.062	3.027	-1.627	-	-	3.725	4.094
45-64	-2.912	-0.315	-0.062	3.304	-2.156	2.215	2.754	3.158	3.646
65+	-2.046	-0.458	-0.062	3.341	-	2.504	2.571	3.097	4.337

S4 Table . Model K: Coefficient estimates for the age-specific model of IPD including Flu, rhinovirus and RSV
