# Supplementary material for: Estimating age-stratified influenza-associated invasive pneumococcal disease in England: A time-series model based on population surveillance data
Source: PLoS Med. 2019 Jun 27;16(6):e1002829. doi: 10.1371/journal.pmed.1002829 (PMC6597037; doi:10.1371/journal.pmed.1002829)
Supplement: S5 Table — IPD, invasive pneumococcal disease; RSV, respiratory syncytial virus. (PDF) [file pmed.1002829.s018.pdf]

Age	α	γ	δ	$log(\psi)$	$log(\tau)$	$log(\theta)$	$log(\zeta)$	$log(\lambda)$	$log(\phi)$
< 5	0.016	0.017	0.003	0.039	-	0.311	1.140	0.011	0.016
$5 - 14$	0.014	0.009	0.003	0.072	0.100	-	-	0.052	0.025
$15 - 44$	0.006	0.006	0.003	0.022	0.066	-	-	0.007	0.001
$45 - 64$	0.014	0.012	0.003	0.018	0.671	0.040	0.051	0.006	0.001
65+	0.004	0.003	0.003	0.011	-	0.028	0.047	0.003	0.001

S5 Table . Model K: Coefficient standard errors for the age-specific model of IPD including Flu, rhinovirus and RSV
